# Extracorporeal Membrane Oxygenation (ECMO) Prediction of Mortality and Integrated Complication with Infection Risk Assessment by Machine Learning: Structured Narrative Review

**DOI:** 10.3390/arm94050069

**Published:** 2026-09-29

**Authors:** Banu Ayse Birlik, Oumayma Hamlaoui, Hakan Tozan, Safa Bhar Layeb, Mohammed Ait El Fqih

**Affiliations:** 1 Department of Medical Services and Techniques, Istinye University, Istanbul 34245, Türkiye; banu.birlik@istinye.edu.tr; 2 College of Engineering and Technology, American University of the Middle East, Egaila 54200, Kuwait; hakan.tozan@aum.edu.kw; 3 Industrial Engineering Center, IMT Mines Albi, University of Toulouse, 81000 Albi, France; safa.layeb@mines-albi.fr; 4 Ingénierie des Systèmes Intelligents, Industrielle et Mécanique (LISIME), ENSAM (École Nationale Supérieure d’Arts et Métiers), Hassan II University of Casablanca, Casablanca 20360, Morocco; mohammed.aitelfqih@ensam-casa.ma

**Keywords:** extracorporeal membrane oxygenation, machine learning, risk prediction, clinical decision support, multi-outcome prediction, infection prediction

## Abstract

**Highlights:**

**What are the main findings?**

- There is a potential for the use of ML models in predicting ECMO risk.
  Models have been applied to mortality, complications, decannulation and readmission.
- External validation, calibration, and retrospective studies are still limited.
  The major gap is the absence of a specific ML models for predicting secondary infections.
  Current models tend to be one dimensional and deterministic.

**What is the implication of the main finding?**

- Future ECMO models should be dynamic, time-updated, and multi-outcome.
  Models should include mortality, complications, infection risk, decannulation, and readmission.
- They need external validation, calibration, interpretability, and prospective clinical testing before real clinical use.

**Abstract:**

**Background:** Extracorporeal membrane oxygenation (ECMO) is an extremely complex life-saving treatment that is performed on patients who are in severe cardiac and/or respiratory failure. Even with all the technological progress, ECMO is a procedure with high mortality and significant morbidity, making it important to predict the risks of ECMO in a timely and accurate manner. **Objective:** To offer a narrative review of the machine learning (ML) based risk prediction models currently used for ECMO, with particular focus on multi-outcome prediction—mortality, complications and infections. **Methods:** Structured narrative review of relevant literature was undertaken in PubMed, Web of Science, Scopus and IEEE Xplore from 2000 to August 2025. Eligible studies comprised machine-learning or artificial-intelligence algorithms to predict clinically important ECMO outcomes and in ECMO-supported patients. Studies that did not include conventional statistical methods, non-ECMO patient populations, or articles that presented editorials, commentaries, or articles lacking clinically relevant prediction outcomes were excluded. Titles and abstracts were screened; full text assessed for eligibility and extracted data organized using the CHARMS framework. **Results:** 25 studies were included, and these were divided into four areas, namely, mortality prediction, complication prediction (neurological, bleeding, thrombosis), requirement for ECMO and process management, and hospital readmission. In all these areas, ML models consistently showed better performance than traditional risk scores, picking up the high-dimensional interactions and nonlinear relationships. But the majority of current models are outcome-specific and use mostly static or pre-ECMO data. Of note, no potentially eligible ECMO-specific machine-learning model to predict incident secondary infection during ECMO support was found despite the impact of infection on outcomes, length of stay, antimicrobial usage, and health-care costs. **Conclusions:** The current evidence suggests that there is a shift in approach from traditional scoring methods to predictive approaches using ML. However, there are still key missing elements such as the lack of incorporation of dynamic physiological data, the lack of incorporation of immunological markers, and the lack of multi-outcome predictive approaches. These limitations need to be overcome to build clinically actionable decision-support systems. ML-based models are very promising in improving risk stratification in ECMO. Other studies should be conducted to create multi-outcome prediction models which incorporate mortality, complications, infection risk and real-time clinical data. These models could be integrated into routine ECMO practice, into electronic health records or ECMO monitoring systems, to generate time-updated and interpretable risk alerts to inform early intervention, antimicrobial stewardship, and resource planning.

## 1. Introduction

Extracorporeal membrane oxygenation (ECMO) is an advanced life support modality that is used for patients whose hearts and/or lungs are failing despite the use of conventional cardiac and/or respiratory life support. The principle behind ECMO is the concept of oxygenating blood and eliminating carbon dioxide (CO_2_) outside of the body, providing a temporary means of sustaining the heart, lungs or both [1]. ECMO is an important therapeutic modality in contemporary intensive care medicine, originally developed in the 1970s from cardiopulmonary bypass. It is now being used in clinical situations like refractory cardiogenic shock, severe postoperative cardiac complications and acute life-threatening respiratory failure. ECMO is often used as a Bridge to Recovery to organ transplantation or other advanced therapeutic interventions, by quickly restoring systemic oxygenation and end-organ perfusion [2]. Although ECMO has life-saving potential, it is a very complex and resource-intensive procedure. To implement it effectively there must be very close collaboration within a multi-disciplinary group that includes intensivists, cardiologists, cardiac surgeons, pulmonologists, perfusionists and specialized critical care nurses. Such a multidisciplinary approach is especially important in veno-arterial ECMO (VA-ECMO) where maintaining haemodynamic stability, adequate organ perfusion and avoiding severe complications are key outcomes for patients in this multidisciplinary setting [3]. Today, two main types of ECMO are available for clinical treatment: veno-venous (VV-ECMO) and veno-arterial ECMO (VA-ECMO) [4,5].

### 1.1. Veno-Venous Extracorporeal Membrane Oxygenation

Veno-venous ECMO is usually used in patients who have severe but potentially reversible respiratory failure. This is not a direct cardiac support configuration as it does not support the heart but rather allows effective oxygenation and removal of carbon dioxide whilst the lungs are being assisted. VV-ECMO has been proven to enhance survival in a select group of patients with acute respiratory distress syndrome (ARDS) [6]. However, large morbidity is found with VV-ECMO, with neurological complications being one of the most common causes of both short- and long-term complications. Neurological complications have been reported between 4 and 13% and include brain death, intracerebral hemorrhage (ICH), ischemic stroke and seizures [7].

Recent machine-learning studies have started to focus on neurological injury and neurological outcomes in ECMO patients but are still limited by different configurations of ECMO, timing of predictions, validation approaches, and lack of dynamic physiologic and circuit variables [8,9,10,11,12,13,14].

Several risk factors have been described, including pre-ECMO cardiac arrest, hyperbilirubinemia, rapid changes of arterial carbon dioxide tension (PaCO_2_) around the time of cannulation and prolonged coagulation times, but there is still a challenge in accurate prediction of adverse neurological outcomes in VV-ECMO patients [8].

### 1.2. Veno-Arterial Extracorporeal Membrane Oxygenation

Veno-arterial ECMO is indicated for patients needing cardiac or combined cardiopulmonary support. It can be life-saving in severe cardiogenic shock, hemodynamic collapse following acute myocardial infarction and refractory circulatory failure following cardiac surgery. VA-ECMO is, however, associated with high mortality and many complications and patient selection is a key component to achieving clinical success.

Parameters used in clinical machine learning (ML) prediction models for ECMO include age, lactate, arterial pH, blood pressure, vasoactive-inotropic support, renal function markers (creatinine and urine output), bilirubin, coagulation markers, platelet count, hemoglobin, oxygenation markers, ventilator markers and ECMO circuit markers (pump flow). These variables are clinically relevant as they indicate circulatory instability, tissue perfusion, organ dysfunction, coagulation status and intensity of ECMO support [3,9,13,14,15,16,17].

Most current ECMO risk prediction is based on traditional clinical scores and static baseline variables. While these tools can be used to perform bedside risk stratification, they have significant drawbacks. They tend to be single outcomes, poorly generalizable across the various ECMO configurations and clinical contexts, and fail to adequately include the evolving physiological and laboratory and circuit data during ECMO support. Such restrictions make it reasonable to consider machine-learning methods, which can model nonlinear interactions and incorporate high-dimensional, time-varying data to improve the ability of risk prediction models to be more personalized [9,15,18,19,20,21,22].

### 1.3. Outcomes and the Current Landscape

In spite of significant technological progress, ECMO-supported patients still have unacceptably high mortality rates at hospital discharge. Mortality rates have been reported to be 59–69% in neonates, 50–64% in pediatric patients and 45–57% in adults [23]. These statistics highlight the ongoing issues of patient selection, complications, and prognosis in ECMO treatment. While a number of studies have examined risk factors for brain injury in ECMO-supported patients, few studies have developed predictive models to predict neurological injury during ECMO. This is a very important deficiency because neurological complications with ECMO have proven to be strongly linked to mortality and long-term morbidity. Identifying patients at high risk for neurological injury early will permit timely intervention and a personalized approach to prevent the injury, thus improving clinical outcomes. Therefore, there is an urgent need for reliable risk prediction models to identify patients early who are at risk of developing ECMO-associated brain injury. Several prognosis prediction models have been developed to predict survival of VV-ECMO patients; nonetheless, the prediction accuracy has been relatively low. Importantly, ECMO-specific scores and general severity of illness scores have shown poor discrimination in COVID-19–related respiratory failure patients on ECMO [24]. Furthermore, these scores tend to be created using traditional statistical modeling methods, and are not always adaptable to different datasets, nor are they always generalizable between different patient populations or clinical contexts. There are three most frequently used prognostic scores in VA-ECMO practice [18,19,20]:

The SAVE score (survival after VA-ECMO) is a pre-ECMO clinical scoring system that utilizes pre-ECMO factors including age, underlying cardiac pathology, presence of organ failure, blood pressure and serum lactate levels to estimate survival probability. First the ENCOURAGE score was designed for patients with cardiogenic shock after AMI and includes parameters such as age, serum lactate, serum creatinine, use of inotropes and arterial pH. Second the REMEMBER score is based on variables collected in the preoperative and intraoperative period and predicts mortality in VA-ECMO patients after coronary artery bypass grafting (CABG).

**Figure 1:**
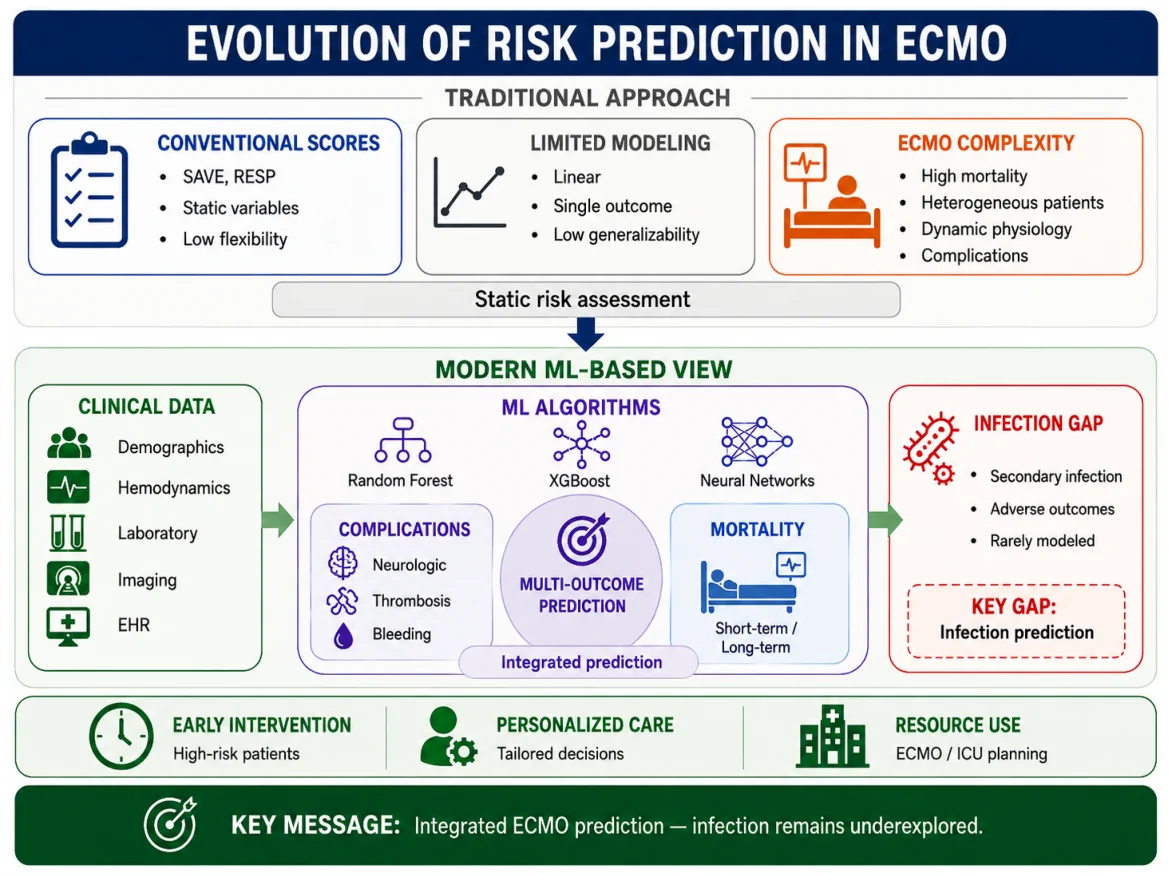
Evolution of ECMO risk prediction static scores to integrated machine learning; gap: infection modelling.

While these scores provide clinical utility in guiding patient selection, they are subject to several important limitations [9]: First, they do not account for continuously acquired hemodynamic and physiologic data collected during ECMO. Second, their predictions mostly apply to in-hospital mortality and patients who are bridged to heart transplantation (HT) or to ventricular assist device (VAD) implantation are usually classified as “survivors,” even though there is no myocardial recovery. Thirdly, there are significant interinstitutional differences in ECMO practices which affect the generalizability of these scoring systems. Traditional statistical methods are still very much applicable in prediction and inference, provided they are properly specified, penalized, validated and calibrated. Some of these ML approaches, however, might provide flexibility to model complex interactions, nonlinear relationships, and high-dimensional data in dynamic ECMO situations. In this context, machine learning (ML) has proven to be a powerful alternative, with the ability to model complex patterns in large-scale datasets, optimize parameters, and take advantage of streams of incoming data to improve predictive accuracy. Some ML techniques are capable of modelling non-linear relationships and can cope with many variables compared to conventional regression techniques. However, sample size requirements are not ignored in ML methods and complex algorithms may require larger datasets and a number of outcome events large enough to produce stable, generalizable and transportable predictions [15,21,22,25]. One advantage of some of the ML methods is that they allow for automatic feature selection and the ability to model interactions between predictors. This is not unique to ML, however, as penalized regression, shrinkage methods, and other statistical methods can also help reduce the number of variables and deal with overfitting [9]. A large Extracorporeal Life Support Organization (ELSO) registry analysis of 23,812 VA-ECMO patients showed that the ML models significantly outperformed the SAVE score for predicting in-hospital mortality (AUC 0.80 vs. 0.61) [15]. Taken together, these data suggest that ML can be a powerful tool to predict both mortality and severe clinical courses, complications, and process-related outcomes in the context of VA-ECMO and VV-ECMO. While traditional risk scores are based on a small number of known variables, ML algorithms can analyze thousands of data points at once, learn the complex relationship between variables and make individualized risk predictions. It is important to note that any parameters measured continuously during ECMO, such as mean arterial pressure, cardiac output and oxygen saturations, measure the patient’s physiology in real time. The use of these dynamic data streams in predictive models could significantly enhance discriminative power and risk estimation and could be more aligned with the clinical state of ECMO patients as it changes. A graphical overview of the development of risk prediction in ECMO and how machine learning is becoming more prominent in multi-outcome prediction is given in Figure 1.

The novelty of this review is that it is integrated—predicting a number of clinically relevant ECMO outcomes, not just mortality. In particular, this review aims to bring together evidence regarding ML models for mortality, neurological complications, bleeding, thrombosis, ECMO requirement and process management, readmission, and risk of infection. A special interest lies in the recognition of the underdeveloped role of secondary infection prediction and the development of decision-support models with multiple outcomes and dynamics. The aim of this review is to summarize current use of ML to predict ECMO, compare the prediction domains in terms of model performance, validation, limitations, and clinical applicability, identify methodological and clinical gaps in the existing literature, and propose future directions for integrated, time-updated, and clinically actionable ML models for ECMO care.

## 2. Methods

### 2.1. Literature Search and Study Selection

To provide a structured narrative synthesis of current literature on machine learning (ML)–based predictive modeling in patients receiving extracorporeal membrane oxygenation (ECMO) and its specific focus on clinically important outcomes including mortality, thrombosis, infection, neurologic complications, process management and hospital readmission, Four major electronic databases—PubMed, Web of Science, Scopus, and IEEE Xplore—were searched to provide as much comprehensive literature as possible. The time frame from which the search was conducted was 1 January 2000 to 1 August 2025, and the geographic location had no boundaries. The results of all four databases are as of 1 August 2025. Controlled vocabulary and free-text terms related to ECMO, machine learning, and prognostic modeling were used in the search strategy. The main keywords and their combinations were: “machine learning,” “risk estimation,” “risk modeling,” “mortality,” “operative mortality,” “extracorporeal membrane oxygenation,” “ECMO,” “VA-ECMO,” “VV-ECMO,” “cardiac arrest,” “neurological injury,” “complications,” “thrombosis,” “infection,” “readmission,” “brain injury,” and “prognostic modeling.” The search strategy was developed using a combination of ECMO-related terms, machine-learning terms and outcome-related terms, with Boolean operators in between. The overall search strategy was: (“extracorporeal membrane oxygenation” OR “ECMO” OR “VA-ECMO” OR “VV-ECMO” OR “extracorporeal cardiopulmonary resuscitation” OR “ECPR”) AND (“machine learning” OR “artificial intelligence” OR “deep learning” OR “neural network” OR “random forest” OR “XGBoost” OR “support vector machine” OR “predictive model” OR “risk prediction” OR “prognostic model”) AND (“mortality” OR “survival” OR “neurological injury” OR “brain injury” OR “bleeding” OR “thrombosis” OR “infection” OR “readmission” OR “ECMO requirement” OR “decannulation”). Search syntax was modified as per the needs of each database.

The same structured search was used to identify infection-related studies, using infection-related terms within the outcome search block. For screening and data extraction, studies were deemed to be infection-relevant studies if they assessed infection as a prediction target or if they reported infection as an ECMO-related complication or infection related variables relevant to future prediction-model development. The following infections were considered infection outcomes: bloodstream infection, ventilator-associated pneumonia, urinary tract infection, surgical-site infection, nosocomial infection, secondary infection while receiving ECMO therapy, and post-decannulation infection, as soon as it was reported.

The studies were eligible if they met the following inclusion criteria: (1) the study population included ECMO patients or patients considered for ECMO support; (2) ECMO configuration included VA-ECMO, VV-ECMO, ECPR, or mixed ECMO support; (3) adult, pediatric, neonatal, or mixed-age patient population was eligible; (4) study design was original clinical prediction-model study (including retrospective cohort studies, prospective cohort studies, registry-based studies, database-based studies, model-development studies, or model-validation studies); (5) the study used at least one machine-learning or artificial-intelligence algorithm for prediction; and (6) the prediction target was clinically relevant to ECMO care: ECMO requirement, mortality, neurological injury, bleeding, thrombosis, infection, decannulation readiness, process-management outcomes, or hospital readmission. Studies were included regardless of whether they compared the machine-learning model to a traditional statistical model or clinical risk score; comparator models were extracted and discussed when reported. The minimum reporting requirements for inclusion were a clearly identifiable population of patients with ECMO, a clearly defined prediction target, a clearly described machine-learning or artificial-intelligence algorithm, and at least one reported measure of model performance or validation.

Studies were excluded if they did not contain ECMO-supported or ECMO-evaluated patients, lacked a machine-learning component, lacked a clinically relevant outcome of ECMO prediction, were not original research articles, were not in English, or were editorials, commentaries, narrative reviews, systematic reviews, conference abstracts with inadequate methodological detail, or lacked model-performance information.

Studies that predicted the need for ECMO in a larger group of critically ill, postoperative, or COVID-19 patients were only included if the target prediction was directly related to ECMO decision making. These studies were considered to be a separate pre-ECMO triage and ECMO-requirement prediction category, separate from studies that predict outcomes in patients already undergoing ECMO support.

Studies that used only conventional statistical methods without machine learning methods were excluded. To identify potentially eligible studies, the titles and abstracts were first screened by the lead reviewer and then by a second reviewer. The full articles were evaluated for inclusion based on the inclusion criteria. Eligibility was discussed amongst the review team where there was any uncertainty until a consensus was established. The lead reviewer screened titles and abstracts and verified with second reviewer. All full texts that had the potential to meet eligibility criteria were evaluated by the two reviewers and disagreements discussed with the review team until consensus was reached. The lead reviewer extracted data using the CHARMS framework, which was then checked by another reviewer. The review team used a methodological quality appraisal based on PROBAST for predefined domains, and consensus was used to reach a judgment in cases of unclear judgment. Information not reported in the original studies but required by the ICMR’s guidelines for data reporting (such as missing data) was treated as “not reported” and not inferred. Studies were only included if they employed an artificial-intelligence or machine-learning algorithm to forecast a clinically relevant outcome related to ECMO. Studies not including ECMO-supported patients, non-English studies, editorials, commentaries and articles without clinically relevant prediction outcomes and studies using only conventional statistical methods without a machine-learning component were excluded. After screening the titles and abstracts, full-text was reviewed for all eligible studies. Twenty-five publications were included based on the inclusion criteria. Data was extracted using the Checklist for Critical Appraisal and Data Extraction for Systematic Reviews of Prediction Modelling Studies (CHARMS) framework that is frequently used to structure the appraisal of prediction modelling studies. The CHARMS checklist has been modified by the authors to meet the needs of this review. This study was developed as a structured narrative synthesis, and did not follow all PRISMA guidelines. There was no formal prespecified review protocol registered in PROSPERO (or other public review registry). The authors established the review objectives and eligibility criteria, search strategy, and outcome domains prior to evidence extraction, but did not create a registered protocol since this is a structured narrative review, rather than a formal systematic review. Therefore, a full record count PRISMA-style flow was not used to describe the selection process. The title, author, year of publication, journal/source data, as well as the DOI (if available), were analyzed and compared to identify duplicate publications. Records were initially screened by title and abstract and subsequently, full-text assessment of potentially eligible studies. The articles were excluded if they did not report clinically relevant prediction outcomes, were review articles, editorials, commentaries, did not use machine-learning or artificial-intelligence methods, or did not include ECMO-supported patients, or did not report ECMO-supported patients.

Data extraction was performed to obtain data that were relevant to prediction-model studies. Captured data included author, year of publication, country, study design, data source, clinical context, ECMO population, ECMO configuration, age group, sample size, prediction time point, target outcome, candidate predictors, algorithm (machine learning or artificial intelligence), comparator model, validation approach, model-performance metrics, calibration reporting, explainability method, feature-selection approach, missing-data handling, and potential clinical applicability when reported.

**Figure 2:**
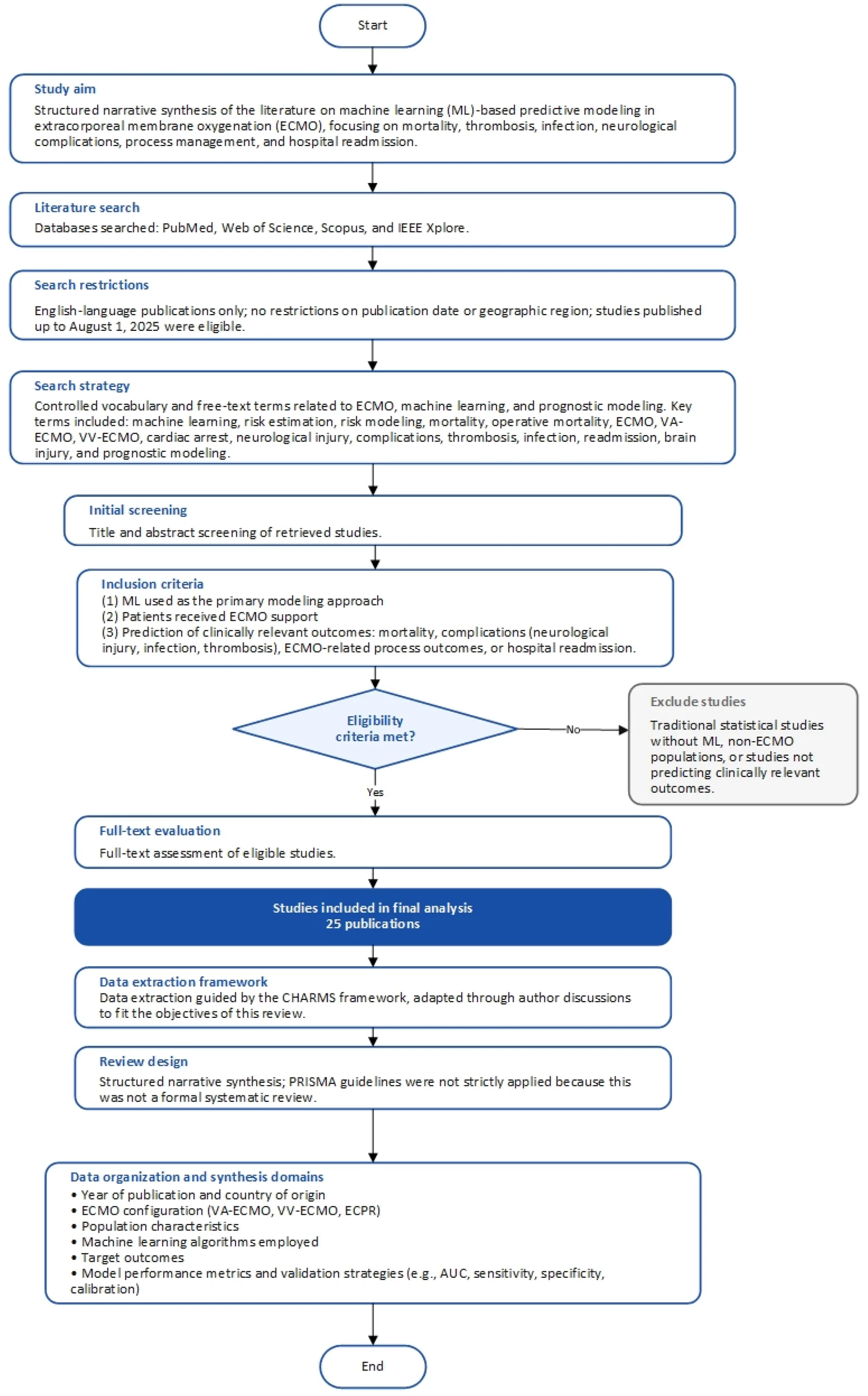
The methodological framework for study identification, eligibility assessment, and evidence synthesis for machine learning–based prediction of ECMO risk.

A flowchart describing the literature identification, selection and synthesis is presented by Figure 2.

Table 1 details the full search strategies used in individual databases, including search fields, Boolean operators, controlled vocabulary, truncation, language and date restrictions, and/or database-specific adaptations.

References published after 1 August 2025 were not included in the structured evidence synthesis, study count or flow diagram and were cited only if relevant to provide contextual information or recent background information. In addition, a focused literature review of recent literature (last 10 years) about ECMO machine learning was conducted prior to resubmission to ascertain if any new publications had altered the interpretation of the key findings, especially the infection-prediction gap. Only post-cutoff studies conducted recently were used to provide context to the Discussion and did not affect the pre-established structured evidence synthesis and studies included.

### 2.2. Data Extraction and Evidence Synthesis

Data were extracted on a proforma sheet, which was created based on the CHARMS framework. Extracted variables comprised author, year of publication, country, study design, source of data, clinical context, ECMO population, ECMO configuration, number of patients, time point of prediction, target outcome, candidate predictors, machine-learning algorithm, comparator model, strategy for validation, performance metrics, reporting of calibration, explainability method, feature-selection approach, missing data treatment, and clinical applicability if provided. The review team confirmed the information obtained from the publications. Prior to resubmission, study titles, publication years, number of patients, ECMO patient populations, outcomes, performance data and reference citations were manually compared and adjusted as needed. If information was not available or not reported in the original study, it was not inferred and recorded as “not reported”. A structured framework for data extraction based on CHARMS, was used to extract relevant information for each eligible study. Extracted data were the study design, the data source, the clinical context, the year and country of publication, the number of patients, the description of patients, the ECMO setup, the time of prediction, the binary outcome, the candidate predictors, the machine learning algorithms, the comparator models, and the stated validation methods. Specific focus was placed on whether the models had incorporated pre-ECMO variables or data obtained during ECMO support, or continuously monitored physiological parameters, laboratory measurements, imaging characteristics or a combination of these data sources. Where reported, information on feature-selection techniques, class-imbalance management, model calibration, explainability methods and handling missing data were also taken into consideration.

Prediction horizons were also extracted and interpreted, when reported, as a model that predicts deterioration, complications or need for ECMO several hours or days before the event is different in its clinical usefulness to a model that produces a prediction shortly before or at the time of the event.

Values of variables collected subsequent to ECMO initiation were interpreted based on the time of collection and their clinical purpose; variables collected after outcome development or as an outcome of ECMO therapy were interpreted with caution as they might be clinically uninformative or leakage from the target.

All measures of model performance provided in the original studies, such as area under the receiver operating characteristic curve, area under the precision–recall curve, sensitivity, specificity, accuracy, precision, calibration measures and Brier scores were used to assess the model performance. Each study reported its validation approach and these were categorized as internal validation, cross-validation, holdout validation, temporal validation and external validation. These approaches to validation were not regarded as equal levels of evidence: Cross-validation and random holdout validation were interpreted as internal validation approaches, while temporal, geographic, and independent institutional validation were interpreted as stronger external or transportability validation approaches. Where feasible, the performance of machine learning model(s) was compared to conventional statistical methods, existing ECMO risk scores and clinician judgement. A pooled analysis (meta-analysis) was not possible, as the included studies were not sufficiently comparable. The most important sources of heterogeneity were ECMO configuration (VA-ECMO or VV-ECMO or ECPR or mixed cohorts), age group, clinical indication, prediction task, prediction time point, outcome definition, prediction horizon, predictor set, algorithm type, comparator model, validation strategy and performance metric. Even studies seemingly reporting the same endpoint like mortality had different definitions (in-hospital vs. 90-day mortality, severe clinical course vs. survival to discharge), different study populations and different validation designs. Hence, results were narratively synthesized in clinically distinguishable prediction domains, and not quantitatively pooled.

The evidence obtained was descriptively summarized and organized in clinically relevant prediction domains. Review target population, time of prediction, target outcome, and intended clinical use were specified in each domain. They were: prediction of ECMO requirement before cannulation, mortality or severe clinical course after cannulation, neurological, bleeding, thrombotic and infectious complications during ECMO support, readiness for decannulation during weaning, and readmission or healthcare utilization after ECMO. Each study was compared in terms of methodological design, predictive performance, validation strategy, interpretability and potential clinical applicability for each domain. This domain-based structure was employed to prevent the mixing of clinically different prediction questions and to make the application of each model type to support decision-making at different points along the pathway of care for ECMO understandable.

Studies were not construed as a general category of machine-learning performance for synthesis. Rather, each study was dedicated to a single clinical prediction task: (1) to determine who would require ECMO; (2) to predict prognosis prior to ECMO cannulation or shortly thereafter; (3) continuously updated prediction of ECMO requirement; (4) prediction of complications during ECMO support, such as neurological, bleeding, thrombotic, or infectious; (5) to predict readiness for weaning from ECMO; and (6) to predict post-discharge outcomes, including readmission or healthcare utilization. These categories were used for interpreting findings, as the populations, time points for prediction, the target outcomes, predictor variables, and planned clinical actions varied across tasks.

### 2.3. Methodological Quality and Risk-of-Bias Assessment

The methodological quality and risk-of-bias assessment was conducted using the available evidence. To support the narrative evaluation of methodological quality and applicability, a methodological appraisal of the included prediction studies was done using the PROBAST methodology [26]. The assessment was conducted on the four primary PROBAST domains: Participants, Predictors, Outcome and Analysis. A structured appraisal guide, the PROBASTdomains, was used to aid the narrative interpretation of the included prediction studies as follows: (1) if the population studied was suitable for prediction for the given objective, (2) if the predictors were clearly defined and available at the time of the intended prediction, (3) if the outcome was clearly defined and measured in a consistent manner, and (4) if the statistical or machine-learning analysis was adequately reported.

Key issues relevant for prediction-model studies were also taken into account: sample size, handling of missing data, risk of overfitting, validation strategy, reporting of calibration, model interpretability, and external validation. Low risk of bias, high risk of bias or unclear risk of bias ratings were assessed for each domain, according to information given in the original studies. The applicability issues related to the target population, availability of predictors, and clinical relevance of the outcome to be predicted were also taken into account.

Given the nature of this review, a structured narrative synthesis, the PROBAST assessment was employed to facilitate interpretation of methodological rigor and clinical applicability, rather than to exclude studies from the synthesis. In the original article, the information to support the domain classification was not sufficiently reported, so the domain was classified as unclear instead of inferred.

## 3. Results

The studies included are described along clinically distinct prediction tasks, and not as an overall evaluation of machine-learning performance. This organization was chosen as pre-ECMO triage, early prognosis after cannulation, dynamic prediction during ECMO support, complication prediction, decannulation support, and post-discharge prediction are different clinical decisions and involve different methodological interpretations.

Machine-learning (ML) methods, which can identify complex patterns in high-dimensional clinical information and capture nonlinear relationships between variables, have been recently investigated as clinical decision support tools for ECMO care. This review of studies shows that in the use of ECMO for veno-arterial (VA-ECMO) and veno-venous (VV-ECMO), ML has been applied to a variety of outcomes, such as death, severe clinical outcomes, complications, and ECMO-related process management.

### 3.1. Quantitative Overview of the Evidence

In all, 25 studies were included in this review. These studies were categorized based on their main prediction goal: mortality or severe clinical event (*n* = 7), complications (neurological injury, bleeding, thrombosis) (*n* = 9), ECMO requirement and management (*n* = 8) or hospital readmission (*n* = 1). These categories are based on the predominant outcome measured/described by each study and should not be assumed to be entirely mutually exclusive clinical categories, as some studies included composite outcome measures, or described results pertinent to more than one prediction category. Taken together, these results indicate that in certain prediction problems, ML models could offer discrimination that is superior to conventional risk scores or regression-based methods; nevertheless, there were inconsistencies across outcomes, populations and methods of validation and generalizable superiority has not yet been established. Table 2 provides an overview of the main application areas and the outcome categories for machine learning in ECMO.

The study-level summary of the prediction studies included is provided in Table 3. and includes information such as study design, data source, ECMO population, sample size, prediction target, algorithm type, validation strategy, performance reporting, calibration, explainability, and management of missing data.

### 3.2. Mortality and Severe Clinical Course Prediction

There has been a large amount of literature that has been dedicated to predicting death and severe clinical courses in VA-ECMO and VV-ECMO cohorts. To aid clinical decision-making and in-hospital mortality prediction, Stephens et al. (2023) [15] conducted a large Australian study of 18,167 patients from the ELSO registry who received VA-ECMO. Several different types of ML models were trained with variables including lactate levels, blood pressure, respiratory rate, surgical type and duration of intubation before the start of ECMO. Lactate and age were found to be the most significant factors and the best performance was obtained with a deep neural network (sensitivity 82%, precision 78%) [15]. Likewise, Braun et al. (2023) [27] used unbiased conditional inference tree models for prediction of in-hospital mortality based on pre-ECMO clinical data with an AUC of 0.71. Random forest modeling revealed urine output as one of the important factors associated with mortality, highlighting the prognostic value of renal function in VA-ECMO. The results were supported by Chang et al. (2023) [16], who compared the random forest and logistic regression models in a group of 1342 ECMO patients. In both models, the first day post-ECMO implantation urine output was one of the strongest factors associated with in-hospital mortality. Oliguria was shown to be associated with an increased risk of mortality of about 44% at one year relative to preserved urine output, thus showing its significance in short- and long-term survival. To create an ML model for predicting survival in adult VA-ECMO patients based solely on laboratory data within 48 h of starting VA-ECMO, Ayers et al. (2020) [17] conducted a single-center retrospective study. Based on almost two million laboratory results, 270 summary variables were calculated for each patient. A deep neural network model had an AUC of 0.92 and was significantly better than the SAVE score (AUC 0.65, *p* < 0.01). Interestingly, these factors consistently emerged as the key variables: lactate, age, total bilirubin and creatinine. This study showed that early laboratory data alone could provide very good survival prediction, but stressed the need for multicenter validation. Lee et al. (2023) [28] created ML models to predict 90-day mortality using data from the nationwide multicenter registry in the VV-ECMO setting. However, the XGBoost model and LightGBM models performed better than traditional RESP scores and PRESERVE scores in predicting 90-day and in-hospital mortality. Of these, the XGBoost model had the best positive predictive value (PPV) and area under precision–recall curve (AUPRC), indicating better identification of patients at high risk of death. Importantly, the models were externally validated and used data easily accessible in an electronic health record, making them suitable for clinical implementation.

Further research has taken the use of ML models for mortality prediction a step further to specific high-risk sub-groups. Loyaga-Rendon et al (2022) [29] created an elastic-net model to predict short-term mortality in patients who were treated with VA-ECMO for refractory cardiogenic shock associated with decompensated heart failure or acute myocardial infarction. The model had an AUC of 0.68 in the derivation cohort and 0.72 in the validation cohort, with troponin, renal function, bilirubin, lactate, age, and pH being the most important predictors. Conversely, the results of the study by Kresoja et al. (2024) [30] using the ECLS-SHOCK randomized trial showed that the use of early VA-ECMO alone minimally contributed to the prediction of short-term mortality, as evidenced by the low relative importance of the model for VA-ECMO treatment, which was 1.1% compared with physiological variables. These studies point to the potential of ML-based methods for discriminating between selected mortality and severe clinical-course prediction tasks, compared to traditional statistical methods and risk-scores, across different centres and populations, though not consistently across all validation settings. The evidence, as a whole, suggests that ML models have the potential to improve early patient selection and predict prognosis in ECMO.

### 3.3. Prediction of Extracorporeal Membrane Oxygenation Requirement, Resource Allocation, and Clinical Deterioration

This section focuses on pre-ECMO triage and ECMO requirement prediction studies, which are distinct from studies to develop models among patients already receiving ECMO support, and are thus analyzed separately.

In addition to predicting mortality, machine learning has been used more recently to predict ECMO requirements, inform resource allocation and identify patients who are likely to deteriorate early. The use of these applications is especially important in settings where resources are scarce and rapid, accurate triage decisions are essential, such as during pandemics, in postoperative cardiac care, and in intensive care units. Magunia et al. (2021) [31] tested several ML models with a national electronic registry based on both retrospective and prospective COVID-19 patients in Germany. The authors used an explainable boosting machine (EBM) model to identify the most influential predictors of ICU survival and ECMO requirement. Age, pulmonary dysfunction and inter-hospital transfer were the most important factors predicting subsequent ECMO support. Overall, these results demonstrate the potential of XAI in ML models to aid in the early identification of patients at risk of deterioration and provide a way to guide resource planning for ECMO on a systems level. Bennett et al. (2021) [32] used data from the National COVID Cohort Collaborative (N3C) database for over 1.9 million adults in a multicenter retrospective cohort study. In an analysis of 174,568 SARS-CoV-2–positive patients, the machine learning models trained using the random forest and XGBoost algorithms had good performance throughout the study period predicting severe clinical outcomes, which included patients who required invasive mechanical ventilation, ECMO support, death or hospice discharge (AUC 0.87). Good discrimination was obtained with laboratory and physiological variables acquired early on the first hospital day, and the pH of the arterial blood was the single most important. These results suggest the potential of early risk stratification based on data for downstream ECMO use.

Xue et al. (2023) [33] created a model specifically for prediction of ECMO called ForecastECMO, which predicted the use of ECMO at multiple time points prior to ECMO in critically ill COVID-19 patients. The model was assessed up to 48 h before the start of ECMO and compared with traditional scores like SOFA and PRESET as well as logistic regression-based scores through the use of a gradient boosting tree (GBT) algorithm. ForecastECMO consistently showed good performance with an AUROC of 0.94 in the derivation cohort and 0.95 in the holdout cohort at the clinically relevant 18 h prediction window. These results demonstrate how multi-horizon prediction methods using ML can be beneficial for proactively planning ECMO’s and early interventions in the clinical setting. Morisson et al. (2023) [3] created an explainable ensemble ML model (deep super learner) to predict VA-ECMO implantation in post-cardiotomy low cardiac output syndrome (PC-LCOS) patients in the postoperative cardiac surgery setting. The model was externally validated with perioperative and immediate postoperative data with a good level of performance (AUROC 0.863). Early postoperative arterial lactate, intraoperative vasoactive–inotropic score (VIS), absence of ACE inhibitor therapy, body mass index (BMI) and EuroSCORE II were key predictors. Significantly, this study showed ECMO need can be confidently predicted with data obtained at the end of cardiopulmonary bypass, and thus may be useful for early and informed decision making.

Imaging-based and biomarker-driven ML approaches have provided complementary evidence. Gresser et al. (2021) [34] developed an algorithm (CAD4COVID) for analysing chest CT scans using artificial intelligence (AI) and used it together with SOFA scores to predict the need for ECMO in severe COVID-19. The AUC of the combined model was high (0.91), and the use of imaging-derived features with clinical severity scores could be useful for further improving the early risk stratification and allocation of different ICU resources. Wang et al. (2022) [35] also used LASSO regression and support vector machine models to discover pulmonary vascular resistance (PVR) and the neutrophil-to-lymphocyte ratio (N/L) as strong predictors of ECMO requirement following pulmonary endarterectomy. These results indicate that inflammatory and hemodynamic markers may serve as preoperative markers of higher risk for ECMO requirement after surgery. Most recently Baek et al. (2024) [36] proposed Robust and Interpretable Early Triage Support (RIETS) system, a deep neural network trained with multicenter data from 19 hospitals in South Korea. Using only 11 routinely collected clinical variables, RIETS predicted severe disease progression—critical care admission, mechanical ventilation, ECMO requirement and death—within 15 days of being admitted to hospital. The model had excellent interpretability and clinical relevance and could be used to improve early triage and large-scale planning of ICUs and ECMO resources. These studies indicate that machine-learning systems could be useful at predicting the need for ECMO and clinical deterioration in specific settings and could provide an early-warning and triage function in the right circumstances and with appropriate validation. These models could play a pivotal role in expanding the use of ML in ECMO, extending beyond outcome prediction to proactive resource management and timely care escalation, directly impacting patient safety and operational efficiency.

### 3.4. Prediction of Neurological Complications and Outcomes

One of the worst predictors of death and chronic morbidity in patients supported with ECMO is neurological complications. Over the past few years, a number of studies involving machine learning have specifically focused on the prediction of acute brain injury (ABI) and neurological outcomes, with a particular emphasis on using large registries, high-dimensional physiological data and time-dependent variables. The Extracorporeal Life Support Organization (ELSO) Registry was the origin of two groundbreaking studies by Kalra et al. (2024) [10] that offer the most extensive evidence to date on the use of ML to predict neurological injury in adult ECMO populations. Despite having the same research group with the same data source, the patient population, ECMO setup and focus of analysis varied significantly between the two studies. The first study reviewed 37,473 adults who were undergoing VV-ECMO and found that 7.1% of patients had an ABI. The authors obtained AUROC score values of around 0.70 for the prediction of ABI by using the tree-based ML algorithms such as the random forest algorithm, CatBoost, LightGBM and XGBoost. High center volume, cardiac arrest prior to being placed on ECMO, high pump flow, and high serum lactate during ECMO were the most powerful predictors. These data highlight the importance of pre-ECMO physiological instability and management aspects of ECMO on neurological risk stratification [9].

In a complementary analysis, they screened 35,855 patients treated with VA-ECMO and 10,775 with extracorporeal cardiopulmonary resuscitation (ECPR). The incidence of ABI was higher in VA-ECMO (7.7%) and was significantly increased in ECPR patients (16.5%). The AUROC of VA-ECMO was 0.67 compared to 0.72 for ECPR. Duration of ECMO, high pump flow during the first 24 h, and elevated arterial oxygen tension were all dominant risk factors and advanced age contributed additional risk in the ECPR cohort. Although ECMO configuration–specific modeling strategies are important, the differences in risk profiles and model performance illustrate the methodological differences between the different approaches. Beyond the data from registries in adults, evidence is available for the use of ML for neurological risk prediction. Shah et al. (2020) [11] created a neural network model to predict moderate-severe radiographic brain injury (measured by CT or MRI) in a paediatric cohort. A neural network with 35 variables, including physiological data, organ perfusion indices, acid–base status, use of vasoactive drugs, coagulation parameters, and device-related factors, obtained an AUC of 0.76, which is a better result than both logistic regression (AUC-equivalent sensitivity 62%, specificity 61%) and clinician judgment (sensitivity 39%, specificity 69%). Contribution analysis of the features showed that the physiological variables had the greatest impact on the models while coagulation variables had a lesser impact. Concentrating on neurological results instead of injury alone, Crespo-Diaz et al. (2024) [12] used ML to create a patient selection model for predicting good neurological survival in patients who underwent ECPR following out-of-hospital cardiac arrest (OHCA). Of the 301 patients treated with VA-ECMO, 40% survived neurologically well. Factors found to be important predictors were arrest rhythm at cannulation, intermittent or sustained return of spontaneous circulation, time from arrest to ECMO perfusion, and serum lactate concentration. The model had good discrimination, with an AUC of 0.89 in internal validation and 0.80 in external validation, further validating the use of ML to inform patient selection for ECPR. Deng et al. (2025) [23] also developed and externally validated an ML model to predict brain injury in children receiving ECMO support. The authors compared the performance of ten ML algorithms based on data from the Chinese Society of Extracorporeal Life Support (CSECLS) registry and local hospitals, and found that the best model was a 15-variable random forest model. The model showed high accuracy with an AUC of 0.912 in internal validation and 0.807 in external validation, which showed good generalizability. More recently, Cao et al. (2025) [13] evaluated the utility of high–temporal resolution data in the prediction of ABI in VA-ECMO patients. The authors showed that ML models with high-frequency hemodynamic variables had an AUROC of 0.79 and high specificity (99%) when analysing >3 million data points across 355 patients with an average of 80,000 measurements/patient. Minimum systolic blood pressure on ECMO and pulse pressure variability proved to be the most powerful predictors, highlighting the need for real-time circulatory dynamics in neurological risk. While not only neurological outcomes, the study by Leng et al. (2025) [14] also acknowledges the incremental value of on-ECMO variables. The authors showed that adding on-ECMO physiological data was a useful addition and significantly improved survival prediction when compared with pre-ECMO models or the SAVE score, indirectly highlighting the usefulness of adding dynamic data for neurological and global outcome prediction, using CatBoost models and introducing SHAP-based interpretability. Together, these studies confirm neurologic complications as an important and unique ECMO prediction domain. They also show that models using ML, especially those that include dynamic high-resolution physiology data, can be useful in enriching early risk stratification and prediction of good neurological outcome. The results show that neurological risk prediction is a key area where future research on ML for ECMO and clinical decision-support development efforts should focus because of the devastating consequences of neurological injury on mortality and quality of life.

### 3.5. Prediction of Hematological Complications and Process-Level Management

Patients supported with ECMO are at high risk for hematological complications, especially bleeding and thrombosis. Considering the complex equilibrium between coagulation and bleeding risk, various studies have investigated adopting machine learning (ML) to predict these risks and aid in the management of hemostasis. Abbasi et al. (2020) [37] did a similar early proof of concept study using supervised ML algorithms to predict hemorrhagic and thrombotic events in 44 adult ECMO patients. Various models were compared such as random forest (RF), decision trees (DTs), k-nearest neighbors (kNNs), logistic regression (LR) and recursive feature elimination (RFE). The accuracy of prediction was between 58% and 80% for hemorrhage and 40% and 64% for thrombosis. While this study had small sample size, RF-based feature importance analysis helped identify a number of potential novel predictors that are not necessarily the conventional clinical assumptions, demonstrating the exploratory power of ML in hypothesis generation for coagulopathy related to ECMO. More sophisticated data representations have also been studied in children. Alexander et al. (2023) [38] reviewed 272 patients from 0 to 19 years of age treated with ECMO for cardiac reasons between 2010 and 2020. The goal of the study was to use electronic health record (EHR)–derived data to predict clinically significant bleeding events while maintaining relational information between variables. Besides rebuilding the previously built multivariable logistic regression model using manually curated features, the authors built a graph neural network (GNN) model using a smaller set of structured EHR features. In total 2012 days of ECMO, bleeding events were observed on 14% of the days. The GNN-based approach showed predictive ability that was similar to traditional statistical models and indicated potential for further development of the model to improve accuracy. This suggests that AI models trained directly from EHRs can achieve equivalent (or even better) performance compared with manually designed prediction tools, while also lowering the burden of data curation.

Thrombotic events such as circuit thrombosis, oxygenator dysfunction, hemolysis, venous thromboembolism, and ischemic stroke that occur during ECMO are clinically challenging and challenging to predict using just static risk factors. As noted in more general reviews of thrombosis with ECMO [40]. Longitudinal laboratory, device, and physiological data streams may provide a route to early detection of changing thrombotic risk with ML-based approaches. In addition to complication prediction, ML has also been used for process-level decision making when on ECMO, such as determining readiness for decannulation. Fuller et al. (2024) [39] created a deep learning architecture, the Continuous Evaluation of VV-ECMO Outcomes (CEVVO) model, based on long short-term memory (LSTM) architecture, to provide continuous risk stratification in VV-ECMO patients. The model was developed using data from 118 patients who received care at Columbia University Irving Medical Center between 2016 and 2022, when they continuously monitored a device as well as discrete clinical measurements were taken to assign the patient to a high-risk or low-risk group on a daily basis. CEVVO had significantly better classification performance than contemporary models (*p* < 0.001 and *p* = 0.04) when evaluated using 12 sets of five-fold cross validation. While at the patient level accuracy was still not sufficient to make clinical decisions, the model has the power to capture transient temporal patterns, allowing for effective risk stratification at the population level. These studies as a whole demonstrate that ML-based methods can help to contribute in a meaningful way to the prediction of hematological complications and/or the management of ECMO procedures, including decannulation. Current models, although limited in sample size, single center, and individual-level discrimination, do provide valuable proof-of-concept evidence that dynamic, time-aware ML systems have the potential to improve hemostasis management and enable safer, more individualized ECMO care.

### 3.6. Prediction of Readmission and Long-Term Health Care Utilization

In addition to in-hospital outcomes, machine learning has also been used to predict long-term healthcare utilization and post-discharge trajectories in ECMO survivors. These are increasingly acknowledged as vital measures of patient-centred recovery and health system burden. Balian et al. (2024) [22] used data from the Nationwide Readmissions Database (NRD) from 2016 to 2020 to create an extreme gradient boosting (XGBoost) model to predict non-elective hospital readmission within 90 days after ECMO hospitalization. Of 22,947 patients who underwent ECMO, 19.6% were readmitted during the time frame of this study. The XGBoost model had superior discriminative performance (AUROC 0.64 vs. 0.49; *p* < 0.001), greater precision (mean average precision 0.30 vs. 0.20; *p* < 0.001), and better calibration (Brier score 0.154 vs. 0.165; *p* < 0.001) than traditional logistic regression. SHapley Additive exPlanations (SHAP) were used to improve model interpretability, which revealed that index hospitalization length of stay, heart or lung transplantation, and Medicare insurance status were the most influential predictors of risk of readmission. Chronic liver disease and frailty also became a significant factor in post-ECMO readmission in the subgroup analyses. These findings underscore the importance of considering underlying burden of comorbidity and social determinants of Health. in addition to acute illness severity in ECMO survivors to conceptualize post-discharge vulnerability. Importantly, in this study, the ML-based models can be used to extend the prognostic assessment beyond the ICU and hospital stay, providing actionable information for discharge planning, intensity of follow-up visits and post-acute care coordination. The predictive performance was moderate and the improvement from conventional regression in this study indicated a potential of using readmission-risk prediction as part of ECMO survivorship pathways, but further validation is required.

### 3.7. Findings of the PROBAST-Based Quality Assessment

The PROBAST-based appraisal revealed high heterogeneity in the methodological quality and reporting of the prediction studies included that were based on machine learning. Most studies employed clinically relevant populations of ECMO patients, as well as clinically relevant prediction targets such as death, neurological injury, bleeding, thrombosis, ECMO requirement, decannulation, and hospital readmission. The use of the outcome domains and participants was found to be clinically applicable to ECMO care on the whole. But there were a number of methodological shortcomings. A significant number of studies were of retrospective nature, and a considerable proportion of studies were single-center analyses or registry-based studies with limited reporting of missing-data handling, calibration and of model updating. Not all models were externally validated consistently and some models were only internally validated or cross-validated or holdout tested. A formal numerical frequency was not determined for calibration reporting and external validation because it was inconsistently and incompletely described across the studies, but was interpreted narratively and included in the PROBAST-based appraisal. This led to a greater risk of overfitting and to a less generalizable model performance reported. The most common potential source of bias was the analysis domain. Some of the most frequent issues were the small sample size compared to the number of candidate predictors, problems in reporting hyperparameter tuning, problems in reporting assessment of the calibration, problems in reporting how class imbalance was addressed, and the lack of prospective validation. Explainability of the method was discussed in some of the studies but was not used uniformly throughout the literature, such as SHAP or feature-importance analysis.

In total, the PROBAST assessment revealed that the existing literature on ECMO machine learning is encouraging yet shows methodological diversity. Main strengths: clinically meaningful prediction targets and using routinely available clinical and laboratory predictors. The major drawbacks were the lack of external validation, incomplete reporting of calibration, heterogeneous outcome definitions and inadequate reporting of analytical methods. These results are a testament to the importance of future ECMO prediction studies to adopt standardized prediction-model reporting and validation approaches.

Table 4 summarizes the main findings of the PROBAST-based appraisal.

## 4. Discussion

### 4.1. Principal Findings and Infection-Focused Research Gap

Based on the reviewed evidence, machine learning models hold promise for discrimination in certain ECMO prediction tasks, such as mortality, thrombotic and neurologic complications. The evidence is not, however, consistent in demonstrating the superiority of the traditional risk scores over all outcomes, populations, and validation settings. However, important limitations exist: the vast majority of current models fail to account for secondary infection, which has a direct impact on prognosis, LOS and health care costs in ECMO populations. In several settings studied, the performance of machine learning (ML) models is generally superior to the performance of traditional ECMO risk scores, although the evidence is not uniform and most models have not yet been externally validated and are subject to bias. A deep neural network model (ECMO PAL) was also shown to be superior to SAVE, Modified SAVE and ECMO ACCEPTS in prediction of in-hospital mortality [15], and a proof-of-concept ML model was superior to SAVE in prediction of survival to discharge (AUC 0.92 vs. 0.65) [41]. As for neurologic outcomes, ML models have demonstrated useful discrimination in ECMO brain injury in children (AUC 0.912 internal and 0.807 external) [42] and in acute brain injury in adult VV-ECMO (AUC 0.70), although in the latter case, the ability to discriminate was specifically described as “suboptimal” [43]. Traditional scores continue to be clinically useful but many have only modest discrimination in ECMO populations—for example, the Pediatric ECMO Prediction model was found to have a C-statistic of 0.75 [44], and, when externally validated, the Pediatric Extracorporeal Membrane Oxygenation Prediction model had a C-statistic of 0.64 [45]. High incidence, poor outcome and longer ICU and hospital stays and higher mortality are associated with infection in ECMO patients [42,43,46]. A nosocomial infection was associated with a 32% higher in-hospital mortality and longer ECMO and ICU length of stay in adults [43]. Bloodstream infection was one of the highest predictors among the Pediatric ECMO Prediction model [47]. However, even now the focus of ECMO predictive literature remains more on mortality and neurologic outcomes than on infections, and in the systematic review of ML-based ECMO models, mortality and neurologic outcomes were most frequent with only a small number of models focusing on complications including infection [48]. A major and persistent challenge in the clinical practice of ECMO is infections acquired during ECMO support. Diagnosis and initiation of antimicrobial therapy when appropriate, infection control measures and adjustment of therapy as needed in an extremely complex intensive care settings, must all be performed simultaneously by the clinicians. Common healthcare-associated infections in ECMO patients are bloodstream infections, urinary tract infections, surgical site infections, and ventilator-associated pneumonia infections [42]. The main points are that there is evidence that infections are frequently associated with ECMO and clinically relevant; they are associated with poor outcomes and higher use of resources (ICU and hospital length of stay) and many prediction models for ECMO do not take into account all the prognostic importance of infection [2,42,43,46]. Despite variations in the reported rates of infections from 8% to 64% depending on the ECMO modality, the rates are still higher than other comparable non-ECMO critically ill groups. Of note, there could be a risk of infection after decannulation. Hospital-acquired infections increase the length of ECMO treatment and length of hospital stay and, therefore, create a significant economic burden to the health care system and to the patient. Thus, focusing on prevention and proper infection control in this population becomes imperative. Good infection prevention can help to decrease the incidence of infection, length of stay in hospital and overall use of resources [49,50]. Biomarkers may be potential candidate variables used as an early indicator of the infection and as a stratification for risk. White blood cell-related indices are basic parameters of inflammation and infection, and could play a major role in outcomes of ECMO. Neutrophils are an important part of the innate immune system and may be associated with infection risk and systemic inflammatory response. Lymphocyte levels are a reflection of immune competence and immune dysfunction has been associated with poor outcomes with ECMO [51,52,53,54,55,56]. The eosinophil is not often stressed, but there is growing intensive care literature indicating that the eosinophil may be associated with prognosis, suggesting that it may be considered as a candidate variable in future predictive analyses [57]. In the infection-focused synthesis, evidence was divided into four categories: (1) models that were developed and prospectively tested to predict secondary infection during ECMO placement; (2) studies that aim to predict mortality or prognosis in ECMO patients with infection; (3) studies that included the presence of infection or infection-related biomarkers as a predictor for other outcomes; and (4) studies that described the epidemiology, burden, and/or clinical consequences of infection in ECMO populations. These categories were not considered as equal. We also recognize that bloodstream infection, ventilator-associated pneumonia, urinary tract infection, surgical-site infection, nosocomial infection and post-decannulation infection vary in time to infection, diagnostic criteria, incidence, and clinical impact and that the time to infection, diagnostic criteria, and intended clinical use should be prespecified in any future ECMO infection-prediction model. Within the studies identified, infection was a common complication, predictor, biomarker-related problem, and/or prognostic factor. During our systematic search, we did not find any eligible ECMO-specific machine-learning model that was primarily focused on predicting incident secondary infection in ECMO support. This is a large and clinically significant mismatch. Beyond the impact of infection on survival, there is also an impact on prolonged hospital stay and costs, which highlights the need for predictive strategies targeting infection. Currently available clinical scoring systems have a small number of static parameters and frequently do not reflect the complexity of the ECMO-related pathophysiologies. While most prognostic models have shown better performance based on ML approaches, especially in critically ill populations, current models lack the use of continuous hemodynamic data, inclusion of immunological markers and a wide-ranging phenotypic understanding of clinically interconnected trajectories. In this context, a comprehensive machine learning system that includes important morbidity domains of ECMO such as mortality, thrombosis, neurological complications, and secondary infection is needed in the future. Special focus should be placed on early prediction of the risk of infection, an aspect not sufficiently studied in the existing literature. Future models could be used by clinicians to estimate risks in real time, make decisions proactively, steward antimicrobial use, and make prognostic assessments. To date, the literature has confirmed the potential of ML-based models in various parts of ECMO care, such as outcome prediction, resource planning, and process management. There are, however, important challenges which remain in the area of generalizability, prospective validation, interpretability and ethical integration into clinical workflows. The integration of complex physiological data and infection-related variables into clinically appropriate and maintainable decision support systems for ECMO care, therefore, requires further efforts.

### 4.2. Dynamic, Time-Resolved, and Configuration-Specific Risk Prediction

This review shows that the utility of machine learning in ECMO is not just the use of complex algorithms, but heavily relies on the timing, granularity and clinical context of the data being fed into the algorithm. Lactate, age, renal function, bilirubin, arterial pH, and urine output were repeatedly mentioned as important parameters to be taken into account. These are all routinely collected physiological and laboratory parameters that carry a great amount of information regarding prognosis [15,27,29]. Models that used data collected during ECMO support, however, tended to be more clinically relevant than models that only included information prior to ECMO. Laboratory trajectory early in the laboratory, high-frequency hemodynamics, pump flow variables, PPV and lactate levels on ECMO were examples of other variables that added to information about survival and neurological injury [10,13,14,17]. All of these indicate that the risk for ECMO is not fixed at the time of cannulation but rather is a dynamic process that changes over time as organ functions, circulatory support and treatment intensity vary. However, the use of dynamic ECMO prediction also poses key methodological considerations. Physiological and laboratory variables are collected in an irregular fashion; non-ignorable missingness may be time-dependent and correlated with the severity of illness; treatments like vasoactive support, ventilation adjustments, transfusion, anticoagulation, antimicrobial therapy, or ECMO flow adjustment may be time-dependent, or influence the patient’s risk (and the reverse may hold true). Future models should thus clearly specify prediction windows, be free of temporal leakage, take treatment-confounder feedback into account and adjust risk estimates while the clinical state changes throughout the ECMO support. Future predictive systems should therefore shift from deriving a risk at one time-point to systems which can be continuously updated, being able to re-calculate patient risk throughout the course of ECMO.

The results also highlight the need to tailor prediction models to type of ECMO and clinical outcome of interest. There were differences in the risk factors and model performance among VA-ECMO, VV-ECMO and ECPR populations, especially for neurological complications [9,10,12,23]. The variables that were related to acute brain injury in patients with VV-ECMO were not the same as those found in VA-ECMO or ECPR groups and were influenced by different factors such as underlying diseases, circulatory support, oxygen delivery, cerebral perfusion, and cardiac arrest exposure. The same holds true for bleeding and thrombosis, as static laboratory parameters may be inappropriate to reflect the dynamic relationships between anticoagulation, circuit conditions, inflammatory response and patient physiology [37,40]. Despite the encouraging preliminary studies, the clinical performance of the random forests, neural networks, graph neural networks and other machine learning methods for these complications is still limited due to small sample size and limited external validation. Thus, configuration-specific modelling with longitudinal laboratory, physiological and device-derived data should be combined in the next generation of ECMO prediction tools. This might help to optimize early predictions of neurological, hemorrhagic and thrombotic complications, and decrease the risk of a single general model, applied to clinically diverse ECMO populations. Therefore, this review does not presume that a one-size-fits-all model would apply to VA-ECMO, VV-ECMO and ECPR populations. Future models should be developed, validated, and interpreted based on the type of ECMO configuration, indication, baseline risk, complications, and clinical goals.

### 4.3. Operational Decision Support and Post-ECMO Risk Stratification

The evidence reviewed also shows that decision making about mortality is not the only use of machine learning in ECMO. Multiple models were developed to help predict the need for ECMO, to know when clinical deterioration was imminent, to facilitate the allocation of resources, or to inform process-level decisions like readiness to decannulate [3,31,36,39]. This is a key step towards moving from retrospective outcome estimation to prospective clinical and operational decision support. Machine learning could potentially help clinicians to identify patients that may need earlier escalation of care, as seen in models like ForecastECMO which have high discrimination with information available before severe deterioration or early in the deterioration process [33,36]. The ability to predict the need for ECMO could help mobilize perfusion teams, equipment, intensive care units and specialists in time to care for the patient in a postoperative or critical care environment. However, these models’ clinical utility is not solely related to models’ discrimination but also to calibration, prediction timing, interpretability of the models and the implications of false-positive and false-negative decisions. A model which correctly predicts the need for ECMO, but offers too little time to make clinical decisions may be of little practical use. Future research should then be conducted to determine if the use of the model for triage also enhances the timing of decision-making, the use of resources and patient outcomes when compared to standard clinical assessment.

Machine learning can also be used for the patient management following stabilization, decannulation and hospital discharge. The CEVVO model demonstrates the potential of time-aware architectures in the field of repeated assessment of patients with VV-ECMO during the treatment but its patient-level accuracy was not yet good enough to be used as a stand-alone clinical decision support system [39]. Likewise, the forecasting of 90-day readmission shows the post-hospitalization risk of ECMO is affected by length of stay, health care factors, transplantation status, frailty and chronic disease [22]. The potential applications of these findings are far greater than acute survival and will include the ECMO care pathway such as weaning, discharge planning, follow-up intensity, rehabilitation and post-acute care coordination. But because current readmission models have only a moderate performance, it’s possible that clinical factors are not the best way to capture long-term vulnerability. Other factors such as functional status, socioeconomic factors, medication history, rehabilitation and post-discharge care access might enhance prediction of future health care utilization. Finally, comprehensive or integrated models of outcomes linking acute complications, treatment pathways, decannulation readiness and post-discharge outcomes may serve as a better representation of patients’ recovery as compared with single outcome models.

### 4.4. Critical Comparative Synthesis and Clinical Applicability

Within the studies included, the level of evidence was highly variable for each of the prediction domains. The most mature field of ECMO machine-learning studies was in the prediction of mortality and severe course of illness. Many of the studies in this area were performed on large, registry, multi-center or high-dimensional clinical datasets, and machine-learning models often outperformed conventional ECMO risk scores in terms of discrimination. But it is important to consider these findings with caution, as many of the models are retrospective with a limited number of external validation studies; not all models performed calibration analysis or prospective clinical evaluation.

Models with potential for clinical and operational decision support were also strong for predicting the requirement for ECMO, resource allocation, and early clinical deterioration. They have the primary benefit of allowing for earlier triage, mobilization of ECMO teams, planning of ICU resources and escalation of care. However, their utility is not just based on their model discrimination ability, but also on prediction timing, calibration, interpretability and implications of false positive and/or false negative predictions. But a model with high AUROC may be of limited clinical utility if it does not offer enough time for the clinician to intervene or if the results of using the model are not easy to understand at the bedside.

Therefore, methodological and clinical considerations and contexts of the AUROC values were taken into account, and they were not directly compared between studies with different populations, different prevalence of the condition being predicted, different prediction horizon, different ECMO configuration, different validation method. The performance of a high AUROC in an internal or random holdout test, for example, was not necessarily interpreted as being superior to a lower AUROC reported after external, temporal, geographic or independent institutional validation in a more heterogeneous patient population.

Some studies compared extensively optimized ML algorithms with unupdated clinical scores or with regression models which were minimally specified, so that comparisons between the use of ML models and conventional approaches were also interpreted with caution. In such cases, ML superiority is only apparent as the model development procedure, the tuning, the selection of features and the validation strategy may be different for the two algorithms. Comparisons were thus made based on the model complexity, development, validation, calibration and clinical usefulness, and not just a difference between ML and statistics.

It was also decided to examine calibration, separately from discrimination. If reported in the original studies, calibration-in-the-large, calibration slope, calibration plots, Brier score and recalibration requirements were taken into consideration in the interpretation of the model’s reliability. Studies that reported only AUROC or other discrimination measures, without reporting results from a calibration assessment, were interpreted with caution because a good discrimination result is not necessarily accompanied by a good individual risk estimation result.

In cases of unusual ECMO complications, AUROC was interpreted with caution as it might be less informative in highly imbalanced datasets. When reported, area under the precision-recall curve, PPV, NPV, sensitivity, specificity, and thresholds were deemed important metrics for assessing the clinical usefulness of models used when predicting rare outcomes like infection, thrombosis, bleeding, or neurological injury.

In addition, the usefulness of models was considered in light of the clinical consequences of false-positive and false-negative predictions. A false positive prediction of ECMO need can result in unnecessary mobilization of resources, planning for transfer or escalation of monitoring, whereas a false negative can result in delay of referral or timely consideration of ECMO. Likewise, if the infection is falsely predicted as negative, then the opportunity for timely diagnosis or treatment is lost, and timely diagnosis or treatment of neurological injury, bleeding, or thrombosis may likewise be lost. Similarly, falsely predicting the infection as positive may result in further testing, exposure to treatment, and/or increased clinician workload. Hence, the use of clinically meaningful thresholds must be based on the specific clinical outcome, the benefits of early intervention and the risks of a misdiagnosis.

In this review, the difference between predictive accuracy and clinical effectiveness is therefore made. Most of the included studies assessed model development, discrimination, calibration, or validation; although none tested if the model implementation would lead to better treatment decisions, patient outcomes, antimicrobial use, or resource allocation in the clinical setting. The performance data reported should thus be interpreted as being a demonstration of predictive ability, and not a demonstration of clinical benefit. Future implementation trials are required to assess if the use of ECMO machine-learning models has an impact on decision-making and patient-centred outcomes versus usual care.

Prediction of neurological complications was another domain of importance, but also of great diversity. Moderate discrimination was generally observed with adult models for VV-ECMO and VA-ECMO, with selected pediatric or ECPR focused models demonstrating higher discrimination including external validation. These differences indicate that configuration-specific models of neurological risk prediction might be needed since the mechanisms and predictors of neurological injury vary between VV-ECMO, VA-ECMO, and ECPR populations. Thus, one general model might not be as clinically applicable as models customized for ECMO configuration, patient group, and prediction time point.

Evidence of hematological complications, such as bleeding and thrombosis, was less mature. Previous research demonstrated some valuable proof-of-concept data, but with varying model performance and often small sample sizes, incomplete reporting of model calibration, and limited external validation. These results suggest that more comprehensive data sets across centres are needed and that dynamic coagulation markers, laboratory trends, circuit variables and physiological data may need to be better incorporated to predict bleeding and thrombosis.

The domain least represented was readmission prediction, with one study included. The use of machine learning led to better discrimination than logistic regression, but the overall discrimination was still moderate. This indicates that there may be aspects of post-ECMO outcomes not adequately represented in acute hospital datasets such as frailty, access to rehabilitation, socioeconomic parameters, functional recovery, discharge destination or post-discharge care pathway.

Models which use variables that are routinely collected, give an interpretable output, external validation and make predictions in time to allow actionable clinical decisions are the most promising from the point of view of clinical applicability.

The outputs of explainability, such as SHAP values and feature-importance rankings, were not seen as indicators of causal relationships, and were instead interpreted as indicators of model contribution. Modifiable causal determinants were therefore not included unless there was more clinical evidence of high ranking. The most prominent features in the literature reviewed were the focus on clinically relevant outcomes, the use of larger databases, and an increased interest in dynamic and/or high-dimensional data. The major limitations were the lack of consistent external validation, missing calibration reporting, the different definitions of the outcome, the different prediction time points, and the limited prospective testing. However, institutional disparities in patient selection for ECMO, cannula insertion, monitoring frequency, antimicrobial treatment, EHR systems, and ECMO treatment protocols can also impact model transportability. Hence, external validation of the system across different centers and ECMO programs is necessary, with local validation and recalibration if necessary, in order to implement the system clinically. Importantly, secondary infection is a relevant and large, yet underdeveloped prediction target, and is linked to mortality, LOS, antimicrobial use, and healthcare costs. This is an area where future integrated multi-outcome ECMO prediction models are needed, incorporating mortality risk, neurological complications, thrombosis, bleeding, readmission, as well as risk of infection.

To help present a more critical synthesis than that offered by listing each individual study in sequence, Table 5 summarizes the most prominent domains of ECMO machine learning prediction and their model performance patterns, methodological strengths, and limitations, and potential clinical applicability.

This comparative synthesis reveals that the evidence base is strongest for mortality prediction and ECMO requirement prediction, whereas less work has been done on bleeding prediction, thrombosis prediction, readmission prediction, and secondary infection prediction, and these latter models could benefit from more extensive validation, reporting of clinically relevant model calibration, and more clinically actionable models.

### 4.5. Future Directions for Dynamic Multi-Outcome Prediction Models

Future machine-learning models of ECMO should break away from the notion of a single pre-ECMO prediction, and use dynamic time-resolved clinical variables gathered before and during ECMO support. There are important candidate variables such as consecutive lactate levels, arterial pH, arterial oxygen and carbon dioxide tensions, mean arterial pressure, PPV, vasoactive-inotropic support, urine output, creatinine, bilirubin, coagulation parameters, platelet count, hemoglobin, pump flow, sweep gas flow, oxygenator-related variables, ventilator settings and changes of organ-support requirements. These variables can have a better idea of the changing condition of patients on ECMO than a single baseline measurement.

Biomarkers and immune-inflammatory markers may be considered as candidate variables in future models for secondary infection prediction, a target that remains insufficiently developed in ECMO. These variables should be interpreted according to when they become available. Variables measured before the prediction point may be used as candidate predictors, whereas culture results, antimicrobial exposure, ECMO duration, ICU length of stay, and hospital length of stay require careful timing interpretation because they may occur after infection onset, reflect clinical suspicion, or represent later outcomes. Future infection-prediction studies should therefore prespecify the infection definition, prediction window, and timing of each candidate predictor.

Multi-outcome prediction models for ECMO use are needed and mortality alone should not be a clinically useful outcome. Future models should be developed instead to predict the risk of death, neurological injury, bleeding, thrombosis, secondary infection, decannulation failure, and readmission. These models should be repeated throughout the course of ECMO support and not just make a single prediction at cannulation. Methods with time awareness, such as recurrent neural networks, gradient-boosting models with time-window features, survival models and dynamic ensembles may be of help. An integrated ECMO decision-support system, however, should not be seen as a single model predicting all outcomes in a similar fashion. Mortality, infection, bleeding, thrombosis, neurological injury, decannulation and readmission are all possible at different points on the ECMO pathway and can be interdependent. For instance, death prevents subsequent infection or readmission and readmission can only be assessed in patients who survive discharge. Future systems could thus be needed to include linked outcome-specific models, competing-risk approaches, multi-state models or other time-dependent models, instead of a single model for all outcomes.

Future models should be externally validated in various ECMO setups (VA-ECMO, VV-ECMO and ECPR), in adult, pediatric, neonatal, postoperative and COVID-19 patient cohorts for clinical translation. In future studies, the procedures for calibration, decision-curve analyses, handling of missing data, class imbalances, explainability methods, and updating of the models should be reported. Predictions should be explainable, providing clues to the clinicians whether the prediction is based on organ dysfunction, coagulation changes, markers of infection, hemodynamic instability or variables on the circuit. The steps would help shift ECMO prediction models from retrospective risk classifiers to real-time clinically actionable decision support systems. Optional table to add (if available). These future systems should also reflect the wider healthcare process-management viewpoints and decision-analytic techniques can help formulate complex operational priorities to enable evidence mapping [58].

Table 6 lists variables and biomarkers that could be included in future multi-outcome ECMO machine-learning models.

Future models could benefit from the inclusion of these time-resolved variables; they may increase the accuracy of models to identify shifting risk profiles during ECMO support and to enable earlier, individualized patientspecific clinical interventions.

### 4.6. Limitations

This review has a number of limitations. Most available evidence was either retrospective, model-development or model validation studies, with many of the studies being single-center analyses or registry-based studies with limited prospective evaluation. Secondly, only publications in English were reviewed, which may have led to a language bias. Third, publication bias cannot be ruled out since some studies with positive or more successful machine-learning models could be more inclined to be published as compared to studies with negative or inconclusive findings. Fourthly, there may be a risk of cohort overlap between some studies using large registries or the same data sources, for instance, the ELSO registry; thus, some publications from the same registry should not necessarily be regarded as completely independent evidence unless it is reported that different cohorts were used. Fifth, the various forms of ECMO, patient cohorts, definitions of outcomes, data sources, prediction horizons, and validation strategies employed in various studies prevented direct comparisons and the use of meta-analysis. Furthermore, since the structured search was completed on 1 August 2025, very recent research published in the revision period may not have been included in the formal synthesis, but was added as a target post-cutoff check to ensure that no important changes in the interpretation had occurred. Sixth, several studies did not fully report the process of calibration, missing-data management, class-imbalance management and model updating, which hampers the evaluation of individual risk estimation and the transportability of the model. Seventh, abstract-only or incomplete reporting of evidence may not offer enough methodological information to conduct a complete appraisal of prediction-model evidence. Lastly, few models were prospectively validated or evaluated during clinical use; and the evidence currently available is for predictive ability, not established clinical effectiveness.

## 5. Conclusions

This structured narrative review aims to bring attention to the increasing use of machine learning algorithms for risk prediction in the ECMO setting. Depending on the studies reviewed, ML was used to predict various clinically relevant outcomes such as death, neurologic injury, bleeding, thrombosis, ECMO use, decannulation, clinical deterioration and hospital readmission. In several studies, better discrimination was achieved using ML models compared to traditional risk scores or regression-based models, particularly using large data sets and clinical, laboratory, or dynamic physiological data. But this result was not observed with all prediction tasks and generalizable superiority remains unproven.

But current evidence is diverse and not to be taken at face value. Many studies were retrospective, single-center or registry-based analyses and external validation was not consistently done. Several studies also reported incomplete descriptions of calibration and handling of missing data, class-imbalance handling and updating of models. Furthermore, many existing models are only based on single outcomes, and most of them only include variables before ECMO, which do not reflect the dynamic clinical course of ECMO-supported patients.

One of the primary gaps found in this review is the lack of an eligible ECMO-specific machine-learning model that was primarily developed to predict incident secondary infection during ECMO support identified in our structured search. This gap is clinically relevant as infection during ECMO is known to be associated with poor outcomes, prolonged ECMO runs, prolonged ICUs and hospital stays, additional health care costs, and resource use. Yet, to date, infection is not often utilized as a main prediction end result in ECMO machine learning research.

Future ECMO prediction models should thus shift towards multi-outcome time-updated, integrated decision-support systems. These should be combined models which estimate the risk of death, neurological complications, bleeding, thrombosis, secondary infection, decannulation failure and readmission. They should also integrate clinical data routinely collected in the clinical setting with dynamic physiological, laboratory, coagulation, circuit and infection biomarkers. Future models should be externally validated and well calibrated, be interpretable, and be tested in various ECMO populations and configurations in a prospective manner to support clinical implementation.

To summarize, ML-based prediction models in ECMO have the potential to be used to stratify risk and aid clinical decision-making, but there is yet to be proof of superiority over conventional models in a generalizable way. Existing evidence lacks external validation, calibration reporting, and clear clinical impact, and is limited by heterogeneous populations and outcomes and retrospective study designs. Future directions for research should focus on prospective validation, local model recalibration, workflow integration, and implementation studies to evaluate whether these models would enhance clinical practice or improve ECMO treatment decisions, clinical outcomes, antimicrobial stewardship, and resource management in real-world ECMO practice.

## Figures and Tables

**Table 1:** Complete database-specific search strategies.

Database	Complete Search Syntax and Restrictions
**PubMed**	((“Extracorporeal Membrane Oxygenation”[Mesh] OR “extracorporeal membrane oxygenation”[tiab] OR ECMO[tiab] OR “VA-ECMO”[tiab] OR “VV-ECMO”[tiab] OR “veno-arterial ECMO”[tiab] OR “veno-venous ECMO”[tiab] OR “extracorporeal cardiopulmonary resuscitation”[tiab] OR ECPR[tiab]) AND (“Machine Learning”[Mesh] OR “Artificial Intelligence”[Mesh] OR “machine learning”[tiab] OR “artificial intelligence”[tiab] OR “deep learning”[tiab] OR “neural network*”[tiab] OR “random forest”[tiab] OR XGBoost[tiab] OR “support vector machine*”[tiab] OR “predictive model*”[tiab] OR “risk prediction”[tiab] OR “prognostic model*”[tiab]) AND (“Mortality”[Mesh] OR mortality[tiab] OR survival[tiab] OR “neurological injur*”[tiab] OR “brain injur*”[tiab] OR bleeding[tiab] OR hemorrhage[tiab] OR haemorrhage[tiab] OR thrombosis[tiab] OR thrombotic[tiab] OR infection*[tiab] OR readmission[tiab] OR decannulation[tiab] OR “ECMO requirement”[tiab])). *Limits applied:* English language; 1 January 2000 to 1 August 2025.
**Web of Science**	TS=((“extracorporeal membrane oxygenation” OR ECMO OR “VA-ECMO” OR “VV-ECMO” OR “veno-arterial ECMO” OR “veno-venous ECMO” OR “extracorporeal cardiopulmonary resuscitation” OR ECPR) AND (“machine learning” OR “artificial intelligence” OR “deep learning” OR “neural network*” OR “random forest” OR XGBoost OR “support vector machine*” OR “predictive model*” OR “risk prediction” OR “prognostic model*”) AND (mortality OR survival OR “neurological injur*” OR “brain injur*” OR bleeding OR hemorrhage OR haemorrhage OR thrombosis OR thrombotic OR infection* OR readmission OR decannulation OR “ECMO requirement”)). *Limits applied:* English language; publication years 2000–2025.
**Scopus**	TITLE-ABS-KEY((“extracorporeal membrane oxygenation” OR ECMO OR “VA-ECMO” OR “VV-ECMO” OR “veno-arterial ECMO” OR “veno-venous ECMO” OR “extracorporeal cardiopulmonary resuscitation” OR ECPR) AND (“machine learning” OR “artificial intelligence” OR “deep learning” OR “neural network*” OR “random forest” OR XGBoost OR “support vector machine*” OR “predictive model*” OR “risk prediction” OR “prognostic model*”) AND (mortality OR survival OR “neurological injur*” OR “brain injur*” OR bleeding OR hemorrhage OR haemorrhage OR thrombosis OR thrombotic OR infection* OR readmission OR decannulation OR “ECMO requirement”)) AND PUBYEAR > 1999 AND PUBYEAR < 2026 AND LIMIT-TO(LANGUAGE, “English”). *Limits applied:* English language; publication years 2000–2025.
**IEEE Xplore**	((“All Metadata”:“extracorporeal membrane oxygenation” OR “All Metadata”:ECMO OR “All Metadata”:“VA-ECMO” OR “All Metadata”:“VV-ECMO” OR “All Metadata”:“extracorporeal cardiopulmonary resuscitation” OR “All Metadata”:ECPR) AND (“All Metadata”:“machine learning” OR “All Metadata”:“artificial intelligence” OR “All Metadata”:“deep learning” OR “All Metadata”:“neural network” OR “All Metadata”:“random forest” OR “All Metadata”:XGBoost OR “All Metadata”:“support vector machine” OR “All Metadata”:“predictive model” OR “All Metadata”:“risk prediction” OR “All Metadata”:“prognostic model”) AND (“All Metadata”:mortality OR “All Metadata”:survival OR “All Metadata”:“neurological injury” OR “All Metadata”:“brain injury” OR “All Metadata”:bleeding OR “All Metadata”:thrombosis OR “All Metadata”:infection OR “All Metadata”:readmission OR “All Metadata”:decannulation OR “All Metadata”:“ECMO requirement”)). *Limits applied:* English language; publication years 2000–2025.

**Table 2:** Application domains of machine learning in extracorporeal membrane oxygenation.

Prediction Category	Number of Studies	Representative Outcomes
Mortality or severe clinical course	7	In-hospital mortality, 90-day mortality, discharge success
Complication prediction	9	Neurological injury, bleeding, thrombosis
ECMO requirement and process management	8	ECMO need, resource allocation, decannulation
Readmission prediction	1	Post-ECMO hospital readmission
Total	25	—

*Note:* Studies were categorized according to their primary prediction objective. Some studies included composite outcomes or variables relevant to more than one prediction domain.

**Table 3:** Study-level characteristics of included ECMO machine-learning prediction studies.

Study	Data Source, Population, and Sample Size	Prediction Target, Algorithm, and Validation	Main Performance and Key Limitation
Stephens et al. (2023) [15]	ELSO registry; large Australian VA-ECMO cohort; *n* = 18,167.	Prediction of in-hospital mortality/survival using deep neural network and other ML models; registry- based validation.	Sensitivity 82%, precision 78%; reported better than SAVE. Large registry design, but calibration and prospective implementation were not fully reported.
Braun et al. (2023) [27]	Multicenter VA-ECMO study using pre-ECMO clinical data; *n* = 837 eligible patients, including 679 in the derivation cohort and 158 in two external validation cohorts.	Prediction of in-hospital mortality using conditional inference trees and conditional inference forests; 10-fold cross-validation and external validation in two cohorts.	AUC 0.71. Moderate discrimination; calibration and missing-data handling were not clearly reported.
Chang et al. (2023) [16]	Clinical ECMO cohort; *n* = 1342.	Prediction of in-hospital mortality and longer-term survival; random forest and logistic regression models.	Urine output was one of the strongest features; oliguria was associated with increased mortality. Detailed calibration reporting was not available.
Ayers et al. (2020) [17]	Single-center retrospective adult VA-ECMO study using laboratory data.	Survival prediction using laboratory data within 48 h of VA-ECMO initiation; deep neural network; internal validation.	AUC 0.92 versus SAVE AUC 0.65, *p* < 0.01. Single-center study; multicenter validation was needed.
Lee et al. (2023) [28]	Nationwide multicenter registry of VV-ECMO patients.	Prediction of 90-day and in-hospital mortality using XGBoost and LightGBM; external validation reported.	XGBoost showed better PPV and AUPRC than RESP/ PRESERVE. Calibration and missing-data details were not fully reported.
Loyaga-Rendon et al. (2022) [29]	VA-ECMO patients with refractory cardiogenic shock due to decompensated heart failure or acute myocardial infarction.	Prediction of short-term mortality using an elastic-net model; derivation and validation cohorts.	AUC 0.68 in derivation and 0.72 in validation. Moderate discrimination; limited reporting details in the manuscript text.
Kresoja et al. (2024) [30]	ECLS-SHOCK randomized-trial dataset; cardiogenic shock patients treated with VA-ECMO.	Short-term mortality/ prognostic-factor analysis using ML-based methods.	VA-ECMO treatment had low relative importance of 1.1%. The study focused on prognostic factor importance rather than a stand-alone prediction tool.
Morisson et al. (2023) [3]	Post-cardiotomy low cardiac output syndrome patients in a postoperative cardiac surgery setting.	Prediction of VA-ECMO implantation after cardiopulmonary bypass; explainable ensemble ML/deep super learner; external validation.	AUROC 0.863. Clinical utility depends on early data availability, calibration, and implementation in practice.
Magunia et al. (2021) [31]	Multicenter COVID-19 ICU registry in Germany; critically ill COVID-19 patients.	Prediction of ICU survival and ECMO requirement; explainable boosting machine; retrospective/prospective registry-based analysis.	Age, pulmonary dysfunction, and inter-hospital transfer were important predictors. Broader ICU population, not restricted to ECMO-supported patients.
Bennett et al. (2021) [32]	National COVID Cohort Collaborative database; 174,568 SARS-CoV-2-positive adults from a database of more than 1.9 million adults.	Prediction of severe clinical outcome including invasive ventilation, ECMO, death, or hospice discharge; random forest and XGBoost; multicenter retrospective cohort.	AUC 0.87. Composite outcome; ECMO was included as part of a broader severe-outcome definition.
Xue et al. (2023) [33]	Critically ill COVID-19 patients at risk of ECMO requirement.	Prediction of ECMO use up to 48 h before initiation; ForecastECMO using gradient boosting tree; derivation and holdout validation.	AUROC 0.94 in derivation and 0.95 in holdout at the 18 h prediction window. COVID-specific pre-ECMO triage model.
Gresser et al. (2021) [34]	Severe COVID-19 ICU patients; CT-imaging-based study.	Prediction of ECMO requirement on admission using CAD4COVID imaging features combined with SOFA; validation details not fully reported.	Combined model AUC 0.91. Imaging-based ECMO- requirement prediction in a COVID-19 setting.
Wang et al. (2022) [35]	Pulmonary endarterectomy surgical cohort.	Prediction of postoperative ECMO requirement using LASSO regression and support vector machine.	PVR and NLR were identified as strong predictors. Surgical-specific cohort; calibration details were not fully reported.
Baek et al. (2024) [36]	Multicenter dataset from 19 hospitals in South Korea; hospitalized COVID-19 patients.	Prediction of severe progression within 15 days, including ICU admission, ventilation, ECMO, and death; RIETS deep neural network; development and validation reported.	Good interpretability and clinical relevance. Composite outcome; not limited to ECMO-specific prediction.
Kalra et al. (2024) [10]	ELSO registry; adults receiving VA-ECMO and ECPR; *n* = 35,855 VA-ECMO and 10,775 ECPR.	Prediction of acute brain injury during ECMO; tree-based ML models; registry- based validation.	AUROC 0.67 in VA-ECMO and 0.72 in ECPR. Moderate discrimination; possible overlap with other ELSO-based registry studies.
Kalra et al. (2024) [9]	ELSO registry; adults receiving VV-ECMO; *n* = 37,473.	Prediction of acute brain injury during VV-ECMO; random forest, CatBoost, LightGBM, and XGBoost; registry- based validation.	AUROC around 0.70. Discrimination was described as suboptimal; possible overlap with other ELSO-based registry studies.
Shah et al. (2020) [11]	Pediatric ECMO cohort.	Prediction of moderate-to- severe radiographic brain injury on CT or MRI; neural network compared with logistic regression and clinician judgment.	AUC 0.76. Pediatric cohort; external validation and calibration details were not fully reported.
Crespo-Diaz et al. (2024) [12]	ECPR cohort after out-of-hospital cardiac arrest; *n* = 301.	Prediction of good neurological survival after ECPR using an ML patient-selection model; internal and external validation.	AUC 0.89 in internal validation and 0.80 in external validation. Specific ECPR/ OHCA population.
Deng et al. (2025) [23]	CSECLS registry and local hospitals; children receiving ECMO.	Prediction of brain injury in pediatric ECMO; ten ML algorithms tested; best model was a 15-variable random forest; internal and external validation.	AUC 0.912 in internal validation and 0.807 in external validation. Pediatric setting; broader transportability still requires further study.
Cao et al. (2025) [13]	High-frequency VA-ECMO dataset; *n* = 355 patients with more than 3 million data points.	Prediction of acute brain injury using high-temporal- resolution hemodynamic data; ML models.	AUROC 0.79; specificity 99%. High-dimensional data require careful validation and leakage control.
Leng et al. (2024) [8]	VA-ECMO dataset.	Survival prediction after VA-ECMO using CatBoost with SHAP interpretability; contribution of on-ECMO variables assessed.	On-ECMO variables improved survival prediction compared with pre-ECMO models or SAVE. External validation details were not fully reported.
Abbasi et al. (2020) [37]	Single clinical ECMO cohort; adult ECMO patients; *n* = 44.	Prediction of hemorrhagic and thrombotic events during ECMO; random forest, decision tree, kNN, logistic regression, and RFE.	Accuracy 58–80% for hemorrhage and 40–64% for thrombosis. Very small proof-of-concept study.
Alexander et al. (2023) [38]	EHR-derived pediatric cardiac ECMO data; *n* = 272 patients and 2012 ECMO days.	Prediction of clinically significant bleeding events during ECMO; Graph Neural Network compared with multivariable logistic regression.	Bleeding events occurred on 14% of ECMO days. GNN performance was similar to the traditional model; further development needed.
Fuller et al. (2024) [39]	Columbia University Irving Medical Center; VV-ECMO patients; *n* = 118.	Daily risk stratification and decannulation-readiness prediction; LSTM-based CEVVO model; twelve sets of five-fold cross-validation.	Better classification than contemporary models, *p* < 0.001 and *p* = 0.04. Patient-level accuracy was insufficient for stand-alone clinical decisions.
Balian et al. (2024) [22]	Nationwide Readmissions Database, 2016–2020; ECMO hospitalizations; *n* = 22,947.	Prediction of non-elective hospital readmission within 90 days; XGBoost compared with logistic regression; SHAP used for explainability.	AUROC 0.64 versus 0.49; mean average precision 0.30 versus 0.20; Brier score 0.154 versus 0.165. Only one readmission study; discrimination remained moderate.

Abbreviations: AMI, acute myocardial infarction; AUC, area under the curve; AUROC, area under the receiver operating characteristic curve; AUPRC, area under the precision–recall curve; CSECLS, Chinese Society of Extracorporeal Life Support; ECPR, extracorporeal cardiopulmonary resuscitation; EHR, electronic health record; ELSO, Extracorporeal Life Support Organization; ECMO, extracorporeal membrane oxygenation; ICU, intensive care unit; kNN, k-nearest neighbors; LASSO, least absolute shrinkage and selection operator; LSTM, long short-term memory; ML, machine learning; N3C, National COVID Cohort Collaborative; NLR, neutrophil-to-lymphocyte ratio; OHCA, out-ofhospital cardiac arrest; PPV, positive predictive value; PVR, pulmonary vascular resistance; RFE, recursive feature elimination; SAVE, survival after veno-arterial ECMO; SHAP, Shapley additive explanations; VA-ECMO, venoarterial extracorporeal membrane oxygenation; VV-ECMO, venovenous extracorporeal membrane oxygenation.

**Table 4:** Summary of PROBAST-based methodological quality assessment.

Domain	Main Appraisal Focus	Main Findings in the Included Studies
Participants	Appropriateness of ECMO population, ECMO configuration, and clinical setting	Most studies included clinically relevant ECMO populations, but populations varied across VA-ECMO, VV-ECMO, ECPR, adult, pediatric, and COVID-19 cohorts.
Predictors	Predictor definition, availability, timing, and clinical usability	Most predictors were routinely available clinical, laboratory, physiological, or EHR-derived variables, but prediction time points varied across studies.
Outcome	Clarity, consistency, and clinical relevance of target outcomes	Outcomes were clinically relevant, including mortality, neurological injury, bleeding, thrombosis, ECMO need, decannulation, and readmission, but definitions varied between studies.
Analysis	Sample size, missing data, validation, calibration, overfitting, and model reporting	This was the main source of concern because many studies had limited external validation, incomplete calibration reporting, unclear missing-data handling, and risk of overfitting.
Applicability	Relevance to clinical ECMO decision support	Applicability was promising but limited by heterogeneity in ECMO configuration, data sources, prediction timing, and validation strategy.

**Table 5:** Comparative synthesis of machine-learning prediction domains in ECMO.

Prediction Domain	Model Performance Pattern	Main Strengths	Main Limitations	Clinical Applicability
Mortality or severe clinical course	Generally moderate to strong discrimination; several ML models outperformed traditional ECMO risk scores [15,16,17,27,28,29,30].	Large registry, multicenter, or high-dimensional datasets; routinely available clinical and laboratory predictors; comparison with existing scores.	Retrospective design, inconsistent external validation, limited calibration reporting, and heterogeneous ECMO populations.	High potential for patient selection, prognosis, and early risk stratification.
ECMO requirement and process management	Often strong discrimination, especially for ECMO need and early deterioration prediction [3,31,32,33,34,35,36,39].	Useful for triage, resource allocation, ICU planning, and early escalation of care; some models used explainable approaches.	Clinical utility depends on prediction timing, calibration, interpretability, and consequences of false-positive or false-negative decisions.	High operational value when predictions are available early enough for action.
Neurological complications	Variable performance; moderate discrimination in some adult registry models and stronger performance in selected pediatric or ECPR models [7,8,9,10,11,12,13,14,23].	Clinically important outcome; some studies used large registries or external validation.	Differences between VV-ECMO, VA-ECMO, and ECPR populations limit generalizability.	Promising, but likely requires configuration-specific models.
Bleeding and thrombosis	Variable and less mature evidence; mainly proof-of-concept performance in small or selected cohorts [37,38,40].	Highlights the potential of ML for complex coagulation-related complications.	Small sample sizes, limited external validation, incomplete calibration reporting, and insufficient integration of dynamic coagulation and circuit data.	Moderate potential, but requires larger multicenter datasets and dynamic predictors.
Readmission	Moderate discrimination; ML improved performance compared with logistic regression in the available study [22].	Extends prediction beyond acute survival to post-discharge care.	Only one included study; post-discharge vulnerability may require social, functional, rehabilitation-related, and follow-up variables.	Useful for discharge planning and follow-up intensity, but evidence remains limited.
Secondary infection	Dedicated ML prediction models remain largely absent, although infection is frequently reported as clinically important in ECMO patients [42,43,45,51,52,53,54,55].	Clinically important and strongly linked to outcomes, length of stay, antimicrobial use, and costs.	Major evidence gap; infection is not commonly treated as a primary ML prediction target.	High future value for antimicrobial stewardship, prevention, and resource planning.

**Table 6:** Recommended variables for future dynamic multi-outcome ECMO machine-learning models.

Variable Category	Candidate Variables	Relevance for Future Prediction Models
Hemodynamic variables	Mean arterial pressure, pulse pressure variability, vasoactive-inotropic support, cardiac output, and pump flow	Reflect circulatory instability, perfusion status, and ECMO support intensity [13,14,15].
Respiratory and gas- exchange variables	Arterial oxygen tension, arterial carbon dioxide tension, arterial pH, ventilator settings, sweep gas flow, and oxygenator parameters	Capture respiratory failure severity, gas-exchange adequacy, and circuit-related support needs [10,13,14,39].
Organ-function variables	Lactate, urine output, creatinine, bilirubin, liver enzymes, and renal replacement therapy status	Reflect evolving tissue perfusion, renal function, hepatic dysfunction, and multiorgan failure [14,16,17].
Coagulation and hematological variables	Platelet count, hemoglobin, activated clotting time, activated partial thromboplastin time, fibrinogen, D-dimer, anticoagulation dose, and transfusion exposure	Support prediction of bleeding, thrombosis, circuit clotting, and transfusion-related risk [37,38,40].
Infection and inflammatory biomarkers	White blood cell count, neutrophil count, lymphocyte count, neutrophil-to-lymphocyte ratio, platelet-to-lymphocyte ratio, procalcitonin, C-reactive protein, eosinophil count, temperature trends, culture results, antimicrobial exposure, and duration of invasive devices	Support early prediction of secondary infection, systemic inflammation, immune dysfunction, and antimicrobial stewardship needs [42,43,45,51,52,53,54,55,56].
Treatment and process variables	ECMO duration, cannulation strategy, decannulation readiness, antimicrobial exposure, invasive device duration, ICU length of stay, hospital length of stay, and transfusion exposure	Capture treatment trajectory, procedural risk, infection exposure, and post-ECMO recovery needs [22,39,43,45,49,50].

## Data Availability

The original contributions presented in this study are included in the article. Further inquiries can be directed to the corresponding author.

## Abbreviations

ABI	acute brain injury
ACE	angiotensin-converting enzyme
AI	artificial intelligence
ARDS	acute respiratory distress syndrome
AUC	area under the curve
AUPRC	area under the precision–recall curve
AUROC	area under the receiver operating characteristic curve
CABG	coronary artery bypass grafting
CAD4COVID	computer-aided diagnosis for COVID-19
CEVVO	Continuous Evaluation of Veno-Venous Extracorporeal Membrane Oxygenation Outcomes
CSECLS	Chinese Society of Extracorporeal Life Support
CT	computed tomography
DT	decision tree
EBM	explainable boosting machine
ECMO	extracorporeal membrane oxygenation
ECPR	extracorporeal cardiopulmonary resuscitation
EHR	electronic health record
ELSO	Extracorporeal Life Support Organization
GBT	gradient boosting tree
GNN	graph neural network
ICH	intracerebral hemorrhage
ICU	intensive care unit
kNN	k-nearest neighbors
LASSO	least absolute shrinkage and selection operator
LightGBM	light gradient-boosting machine
LR	logistic regression
LSTM	long short-term memory
ML	machine learning
N/L	neutrophil-to-lymphocyte ratio
N3C	National COVID Cohort Collaborative
NRD	Nationwide Readmissions Database
OHCA	out-of-hospital cardiac arrest
PaCO_2_	arterial partial pressure of carbon dioxide
PC-LCOS	post-cardiotomy low cardiac output syndrome
PVR	pulmonary vascular resistance
RFE	recursive feature elimination
RF	random forest
RIETS	Robust and Interpretable Early Triage Support
SHAP	Shapley additive explanations
SOFA	Sequential Organ Failure Assessment
VA-ECMO	veno-arterial extracorporeal membrane oxygenation
VAD	ventricular assist device
VIS	vasoactive-inotropic score
VV-ECMO	veno-venous extracorporeal membrane oxygenation
XGBoost	extreme gradient boosting

## References

[1] Makdisi G., Wang I. (2015). Extra corporeal membrane oxygenation (ECMO) review of a lifesaving technology. J. Thorac. Dis..

[2] Giordano L., Francavilla A., Bottio T., Dell’Amore A., Gregori D., Navalesi P., Lorenzoni G., Baldi I. (2023). Predictive models in extracorporeal membrane oxygenation (ECMO): A systematic review. Syst. Rev..

[3] Morisson L., Duceau B., Do Rego H., Lancelot A., Hariri G., Charfeddine A., Laferrière-Langlois P., Richebé P., Lebreton G., Provenchère S. (2023). A new machine learning algorithm to predict veno-arterial ECMO implantation after post-cardiotomy low cardiac output syndrome. Anaesth. Crit. Care Pain Med..

[4] Keebler M., Haddad E., Choi C., McGrane S., Zalawadiya S., Schlendorf K., Brinkley D., Danter M., Wigger M., Menachem J. (2018). Venoarterial extracorporeal membrane oxygenation in cardiogenic shock. JACC Heart Fail..

[5] Rao P., Khalpey Z., Smith R., Burkhoff D., Kociol R. (2018). Venoarterial extracorporeal membrane oxygenation for cardiogenic shock and cardiac arrest. Circ. Heart Fail..

[6] Combes A., Peek G., Hajage D., Hardy P., Abrams D., Schmidt M., Dechartres A., Elbourne D. (2020). ECMO for severe ARDS: Systematic review and individual patient data meta-analysis. Intensive Care Med..

[7] Lorusso R., Belliato M., Mazzeffi M., Di Mauro M., Taccone F., Parise O., Albanawi A., Nandwani V., McCarthy P., Kon Z. (2021). Neurological complications during veno-venous extracorporeal membrane oxygenation: Does the configuration matter? A retrospective analysis of the ELSO database. Crit. Care.

[8] Leng A., Shou B., Liu O., Bachina P., Kalra A., Bush E., Whitman G., Cho S. (2024). Machine learning from veno-venous extracorporeal membrane oxygenation identifies factors associated with neurological outcomes. Lung.

[9] Kalra A., Bachina P., Shou B., Hwang J., Barshay M., Kulkarni S., Sears I., Eickhoff C., Bermudez C., Brodie D. (2024). Acute brain injury risk prediction models in venoarterial extracorporeal membrane oxygenation patients with tree-based machine learning: An Extracorporeal Life Support Organization registry analysis. JTCVS Open.

[10] Kalra A., Bachina P., Shou B., Hwang J., Barshay M., Kulkarni S., Sears I., Eickhoff C., Bermudez C., Brodie D. (2024). Using machine learning to predict neurologic injury in venovenous extracorporeal membrane oxygenation recipients: An ELSO registry analysis. JTCVS Open.

[11] Shah N., Farhat A., Tweed J., Wang Z., Lee J., McBeth R., Skinner M., Tian F., Thiagarajan R., Raman L. (2020). Neural networks to predict radiographic brain injury in pediatric patients treated with extracorporeal membrane oxygenation. J. Clin. Med..

[12] Crespo-Diaz R., Wolfson J., Yannopoulos D., Bartos J. (2024). Machine learning identifies higher survival profile in extracorporeal cardiopulmonary resuscitation. Crit. Care Med..

[13] Cao M., Feng S., Ahmed Y., Liu W., Brown P., Kalra A., Shou B., Bezerianos A., Thakor N., Whitman G. (2025). High-granularity machine learning prediction of acute brain injury in patients receiving venoarterial extracorporeal membrane oxygenation. ASAIO J..

[14] Leng A., Bachina P., Liu O., Shou B., Racz C., Giliver D., Shpitser I., Whitman G., Cho S. (2025). Enhancing survival prediction after venoarterial extracorporeal membrane oxygenation using machine learning. ASAIO J..

[15] Stephens A., Šeman M., Diehl A., Pilcher D., Barbaro R., Brodie D., Pellegrino V., Kaye D., Gregory S., Hodgson C. (2023). ECMO PAL: Using deep neural networks for survival prediction in venoarterial extracorporeal membrane oxygenation. Intensive Care Med..

[16] Chang S., Hu N., Wu J., Cheng H., Caffrey J., Yu H., Chen Y., Hsu J., Lin J. (2023). Urine output as one of the most important features in differentiating in-hospital death among patients receiving extracorporeal membrane oxygenation: A random forest approach. Eur. J. Med. Res..

[17] Ayers B., Wood K., Gosev I., Prasad S. (2020). Predicting survival after extracorporeal membrane oxygenation by using machine learning. Ann. Thorac. Surg..

[18] Amin F., Lombardi J., Alhussein M., Posada J., Suszko A., Koo M., Fan E., Ross H., Rao V., Alba A. (2021). Predicting survival after VA-ECMO for refractory cardiogenic shock: Validating the SAVE score. CJC Open.

[19] Hatami S., Hefler J., Freed D. (2022). Inflammation and oxidative stress in the context of extracorporeal cardiac and pulmonary support. Front. Immunol..

[20] Muller G., Flecher E., Lebreton G., Luyt C., Trouillet J., Bréchot N., Schmidt M., Mastroianni C., Chastre J., Leprince P. (2016). The ENCOURAGE mortality risk score and analysis of long-term outcomes after VA-ECMO for acute myocardial infarction with cardiogenic shock. Intensive Care Med..

[21] Saberi-Karimian M., Khorasanchi Z., Ghazizadeh H., Tayefi M., Saffar S., Ferns G., Ghayour-Mobarhan M. (2021). Potential value and impact of data mining and machine learning in clinical diagnostics. Crit. Rev. Clin. Lab. Sci..

[22] Balian J., Sakowitz S., Verma A., Vadlakonda A., Cruz E., Ali K., Benharash P. (2024). Machine learning based predictive modeling of readmissions following extracorporeal membrane oxygenation hospitalizations. Surg. Open Sci..

[23] Deng B., Zhao Z., Ruan T., Zhou R., Liu C., Li Q., Cheng W., Wang J., Wang F., Xie H. (2025). Development and external validation of a machine learning model for brain injury in pediatric patients on extracorporeal membrane oxygenation. Crit. Care.

[24] Supady A., Dellavolpe J., Taccone F., Scharpf D., Ulmer M., Lepper P., Halbe M., Ziegeler S., Vogt A., Ramanan R. (2021). Outcome prediction in patients with severe COVID-19 requiring extracorporeal membrane oxygenation: A retrospective international multicenter study. Membranes.

[25] Valiente Fernández M., Lesmes González De Aledo A., Martín Badía I., Delgado Moya F. (2024). Comparing traditional regression and machine learning models in predicting acute respiratory distress syndrome mortality. Crit. Care Med..

[26] Wolff R.F., Moons K.G.M., Riley R.D., Whiting P.F., Westwood M., Collins G.S., Reitsma J.B., Kleijnen J., Mallett S. (2019). PROBAST: A Tool to Assess the Risk of Bias and Applicability of Prediction Model Studies. Ann. Intern. Med..

[27] Braun J., Sahli S., Spahn D., Röder D., Neb H., Lotz G., Aser R., Wilhelm M., Kaserer A. (2023). Predicting survival for veno-arterial ECMO using conditional inference trees-a multicenter study. J. Clin. Med..

[28] Lee H., Song M., Cho Y., Kim D., Hong S., Jung S., Lim S. (2023). Supervised machine learning model to predict mortality in patients undergoing venovenous extracorporeal membrane oxygenation from a nationwide multicentre registry. BMJ Open Respir. Res..

[29] Loyaga-Rendon R., Fermin D., Grayburn R., Matthew G., Dickinson M., Manandhar-Shrestha N., Trethowan B., Leacche M., Jovinge S. (2022). Predicting short-term mortality in ECMO-supported patients secondary to decompensated heart failure and acute myocardial infarction using machine learning. J. Heart Lung Transplant..

[30] Kresoja K., Zweymer U., Akin I., Rassaf T., Mahabadi A., Lehmann R., Eitel I., Graf T., Schuster A., Skurk C. (2024). Identifying prognostic factors in patients with cardiogenic shock undergoing VA-ECMO treatment: Insights from ECLS-SHOCK using machine learning. Eur. Heart J..

[31] Magunia H., Lederer S., Verbuecheln R., Gilot B., Koeppen M., Haeberle H., Mirakaj V., Hofmann P., Marx G., Bickenbach J. (2021). Machine learning identifies ICU outcome predictors in a multicenter COVID-19 cohort. Crit. Care.

[32] Bennett T., Moffitt R., Hajagos J., Amor B., Anand A., Bissell M., Bradwell K., Bremer C., Byrd J., Denham A. (2021). Clinical characterization and prediction of clinical severity of SARS-CoV-2 infection among US adults using data from the US National COVID Cohort Collaborative. JAMA Netw. Open.

[33] Xue B., Shah N., Yang H., Kannampallil T., Payne P., Lu C., Said A. (2023). Multi-horizon predictive models for guiding extracorporeal resource allocation in critically ill COVID-19 patients. J. Am. Med. Inform. Assoc..

[34] Gresser E., Reich J., Sabel B., Kunz W., Fabritius M., Rübenthaler J., Ingrisch M., Wassilowsky D., Irlbeck M., Ricke J. (2021). Risk stratification for ECMO requirement in COVID-19 ICU patients using quantitative imaging features in CT scans on admission. Diagnostics.

[35] Wang X., Liu X., Liu Z., Zheng X., Yang Y., Zhen Y., Ye Z., Liu P. (2022). Preoperative pulmonary vascular resistance and neutrophil-to-lymphocyte ratio predict the demand of extracorporeal membrane oxygenation after pulmonary endarterectomy. J. Card. Surg..

[36] Baek S., Jeong Y., Kim Y., Kim J., Kim J., Kim E., Lim J., Kim J., Kim Z., Kim K. (2024). Development and validation of a robust and interpretable early triaging support system for patients hospitalized with COVID-19: Predictive algorithm modeling and interpretation study. J. Med. Internet Res..

[37] Abbasi A., Karasu Y., Li C., Sodha N., Eickhoff C., Ventetuolo C. (2020). Machine learning to predict hemorrhage and thrombosis during extracorporeal membrane oxygenation. Crit. Care.

[38] Alexander P., Gauthier M., Deforte S., Geppert J., Ramirez J., Riedl M., Bickel J., Sleeper L., Thiagarajan R., Dufek S. (2023). AI-enabled prediction of bleeding in patients supported on extracorporeal membrane oxygenation. J. Am. Coll. Cardiol..

[39] Fuller J., Abramov A., Mullin D., Beck J., Lemaitre P., Azizi E. (2024). A deep learning framework for predicting patient decannulation on extracorporeal membrane oxygenation devices: Development and model analysis study. JMIR Biomed. Eng..

[40] Nunez J., Gosling A., O’Gara B., Kennedy K., Rycus P., Abrams D., Brodie D., Shaefi S., Garan A., Grandin E. (2022). Bleeding and thrombotic events in adults supported with venovenous extracorporeal membrane oxygenation: An ELSO registry analysis. Intensive Care Med..

[41] Bailly D.K., Furlong-Dillard J.M., Winder M., Lavering M., Barbaro R.P., Meert K.L., Bratton S.L., Dalton H., Reeder R.W. (2021). External Validation of the Pediatric Extracorporeal Membrane Oxygenation Prediction Model for Risk Adjusting Mortality. Perfusion.

[42] Jiang J., Jiang Y., Xu Y., Zhang S. (2026). ECMO-Associated Nosocomial Infections in Adults: Immunopathogenesis and Predictive Modeling Approaches. Front. Med..

[43] Li X., Wang L., Wang H., Hou X. (2022). Outcome and Clinical Characteristics of Nosocomial Infection in Adult Patients Undergoing Extracorporeal Membrane Oxygenation: A Systematic Review and Meta-Analysis. Front. Public Health.

[44] Keane M., Sajan S., Nockels K., Zochios V., Ahmed A., Yusuff H. (2026). Use of Artificial Intelligence to Predict Outcomes in Critically Ill Patients Receiving Extracorporeal Membrane Oxygenation: A Systematic Review. J. Cardiothorac. Vasc. Anesth..

[45] Peña-López Y., Machado M.C., Rello J. (2024). Infection in ECMO Patients: Changes in Epidemiology, Diagnosis and Prevention. Anaesth. Crit. Care Pain Med..

[46] Marcus J.E., Shah A., Peek G.J., MacLaren G. (2024). Nosocomial Infections in Adults Receiving Extracorporeal Membrane Oxygenation: A Review for Infectious Diseases Clinicians. Clin. Infect. Dis..

[47] Bailly D.K., Reeder R.W., Winder M., Barbaro R.P., Pollack M.M., Moler F.W., Meert K.L., Berg R.A., Carcillo J., Zuppa A.F. (2019). Development of the Pediatric Extracorporeal Membrane Oxygenation Prediction Model for Risk-Adjusting Mortality. Pediatr. Crit. Care Med..

[48] Chen S., Liu L., Hu D., Zhu Y., Wu C., Fu H., Wu J., Zhang K., Chang M., Hao T. (2026). Effect of Artificial Intelligence in Extracorporeal Membrane Oxygenation: A Systematic Review and Meta-Analysis. Intensive Crit. Care Nurs..

[49] Hu Z., Peng M., Dong C., Li L., Hu Q., Liao H., Jiang H., Fang X., Zhang Q., Fu B. (2025). Risk factors and economic burden of healthcare-associated infections among patients supported by extracorporeal membrane oxygenation in the ICU: A cohort study from China. Antimicrob. Resist. Infect. Control.

[50] Zeng Y., Liu Y., Zhou S., Chen Q., Zhang Q. (2025). Hospital-acquired infections in neonatal ECMO patients: A systematic review and meta-analysis. Ital. J. Pediatr..

[51] Xu Z., Qi L., Su S., Xu Z., Geng Y., Shi Y., Wang C., Wu J., Liu R. (2025). Neutrophil-to-Lymphocyte Ratio and Platelet-to-Lymphocyte Ratio as Potential Predictors of Nosocomial Infection in Patients Undergoing Veno-Arterial Extracorporeal Membrane Oxygenation: A Cohort Study. PLoS ONE.

[52] Roth S., M’Pembele R., Stroda A., Jansen C., Lurati Buse G., Boeken U., Akhyari P., Lichtenberg A., Hollmann M.W., Huhn R. (2022). Neutrophil-Lymphoycyte-Ratio, Platelet-Lymphocyte-Ratio and Procalcitonin for Early Assessment of Prognosis in Patients Undergoing VA-ECMO. Sci. Rep..

[53] Doo I., Staub L.P., Mattke A., Haisz E., Seidler A.L., Alphonso N., Schlapbach L.J. (2022). Diagnostic Accuracy of Infection Markers to Diagnose Infections in Neonates and Children Receiving Extracorporeal Membrane Oxygenation. Front. Pediatr..

[54] Gillett E.L., Birişci E., Frazier J.B., Stroud M.H., Malone M.P. (2026). Inflammatory Markers for Nosocomial Infections in Pediatric Extracorporeal Membrane Oxygenation: Single-Center Retrospective Cohort, 2017–2022. Pediatr. Crit. Care Med..

[55] Jackson L.B., Sobieszczyk M.J., Aden J.K., Marcus J.E. (2023). Fever and Leukocytosis Are Poor Predictors of Bacterial Coinfection in Patients With COVID-19 and Influenza Who Are Receiving Extracorporeal Membrane Oxygenation. Open Forum Infect. Dis..

[56] Nunez J.I., Uehara M., Patel S.R., Forest S.J., Rochlani Y., Madan S., Sims D.B., Mellas N., Ashley J.E., Rahmanian M. (2025). Changes in Neutrophil-to-Lymphocyte Ratio During Venoarterial Extracorporeal Membrane Oxygenation. ASAIO J..

[57] Wang S., Tao S., Zhu Y., Gu Q., Ni P., Zhang W., Wu C., Zhao R., Hu W., Diao M. (2025). AI-powered model for predicting mortality risk in VA-ECMO patients: A multicenter cohort study. Sci. Rep..

[58] Kılıç Y., Duzdar I., Hamlaoui O., Tozan H., El Fqih M.A. (2026). Surgical Intensity and Specialization Preferences in Healthcare: An Operation- and Process-Management Perspective Using Bibliometric Analysis, Cognitive Mapping and Analytic Network Process (ANP). Healthcare.

